# IL2 Targeted to CD8^+^ T Cells Promotes Robust Effector T-cell Responses and Potent Antitumor Immunity

**DOI:** 10.1158/2159-8290.CD-23-1266

**Published:** 2024-04-09

**Authors:** Kelly D. Moynihan, Manu P. Kumar, Hussein Sultan, Danielle C. Pappas, Terrence Park, S. Michael Chin, Paul Bessette, Ruth Y. Lan, Henry C. Nguyen, Nathan D. Mathewson, Irene Ni, Wei Chen, Yonghee Lee, Sindy Liao-Chan, Jessie Chen, Ton N.M. Schumacher, Robert D. Schreiber, Yik A. Yeung, Ivana M. Djuretic

**Affiliations:** 1 Asher Biotherapeutics, Inc., South San Francisco, California.; 2 Department of Pathology and Immunology, Washington University School of Medicine, St. Louis, Missouri.; 3 Division of Molecular Oncology and Immunology, Oncode Institute, Netherlands Cancer Institute, Amsterdam; Department of Hematology, Leiden University Medical Center, Leiden, the Netherlands.

## Abstract

IL2 signals pleiotropically on diverse cell types, some of which contribute to therapeutic activity against tumors, whereas others drive undesired activity, such as immunosuppression or toxicity. We explored the theory that targeting of IL2 to CD8^+^ T cells, which are key antitumor effectors, could enhance its therapeutic index. To this aim, we developed AB248, a CD8 *cis*-targeted IL2 that demonstrates over 500-fold preference for CD8^+^ T cells over natural killer and regulatory T cells (Tregs), which may contribute to toxicity and immunosuppression, respectively. AB248 recapitulated IL2’s effects on CD8^+^ T cells *in vitro* and induced selective expansion of CD8^+^T cells in primates. In mice, an AB248 surrogate demonstrated superior antitumor activity and enhanced tolerability as compared with an untargeted IL2Rβγ agonist. Efficacy was associated with the expansion and phenotypic enhancement of tumor-infiltrating CD8^+^ T cells, including the emergence of a “better effector” population. These data support the potential utility of AB248 in clinical settings.

**Significance:**

The full potential of IL2 therapy remains to be unlocked. We demonstrate that toxicity can be decoupled from antitumor activity in preclinical models by limiting IL2 signaling to CD8^+^ T cells, supporting the development of CD8^+^ T cell–selective IL2 for the treatment of cancer.

See related article by Kaptein et al. p. 1226.

## Teaser

*Cis*-targeting of IL2 to CD8^+^ T cells shows improved anti-tumor activity and reduced toxicity compared to broadly acting IL2, supporting the evaluation of such a strategy in patients.

## Introduction

High-dose (HD) IL2 was the first immunotherapy to show complete responses in a subset of patients with cancer (1), and recent studies support the continuing utility of IL2 in the modern era. Specifically, objective response rate (ORR) values of over 20% have been observed with HD IL2 monotherapy in immune-checkpoint inhibitor (ICI) failed melanoma and renal cell carcinoma (RCC) patients, comparable with historical response rates in ICI-naive patients (2, 3). In addition, a 70% ORR was observed in a single-arm phase II trial with HD IL2 + anti–PD-1 therapy in RCC, exceeding single-agent benchmarks in that patient population (4). Despite these promising data, IL2’s clinical use remains limited due to life-threatening toxicities and the need for frequent administration.

IL2 is a pleiotropic cytokine that induces both immunostimulatory and immunosuppressive effects mediated by various immune cell types. IL2 signals via a trimeric, high-affinity IL2Rαβγ, and a dimeric, intermediate-affinity IL2Rβγ. IL2Rβγ is expressed broadly, including on CD8^+^ T cells, CD4^+^ T cells, regulatory T cells (Treg), natural killer (NK) cells, and innate lymphoid cells, whereas IL2Rαβγ is expressed constitutively on Tregs and innate lymphoid cells, and transiently on activated T cells (5). In addition to promoting potent Treg signaling, the high-affinity binding of IL2 to cells that express IL2Rα has been implicated in the development of vascular leak syndrome (VLS; ref. 6), a problematic toxicity associated with HD IL2. To reduce Treg stimulation and VLS, several IL2-based therapeutics have been developed that avoid IL2Rα binding, collectively referred to as “not-α” IL2s. To date, these not-α IL2 molecules have not demonstrated a meaningful improvement in clinical efficacy as compared with historical HD IL2 data (7–10). Importantly, although VLS appears mitigated, clinically significant toxicities still occur (7–10). These data suggest that the induction of IL2 signaling broadly in IL2Rβγ^+^ cells is insufficient to drive substantive improvements in IL2’s therapeutic index even while avoiding IL2Rα engagement. Thus, there remains an opportunity to realize the full potential of IL2 therapy.

We speculated that the targeting of IL2 toward CD8^+^ T cells and away from Treg, NK cells, and other innate populations could enhance its therapeutic index. CD8^+^ T cells are critical effectors of antitumor immunity (11–15), and their stimulation with IL2 induces both T-cell proliferation and enhanced effector function (16). In contrast, in preclinical models, NK cells have been shown to contribute to the toxicities of IL2-based therapy and be dispensable for antitumor activity (17–20). In patients, not-α IL2 variants demonstrated a bias toward NK cell over CD8^+^ T-cell expansion in blood, with approximately 7- to 10-fold NK cell expansion and 2- to 4-fold CD8^+^ T-cell expansion at the top doses explored clinically (21–24). Modest clinical activity was seen with these not-α IL2 variants in patients despite these dramatic NK cell increases (21, 22, 25), suggesting that vigorous NK cell expansion is insufficient to drive substantial clinical efficacy. Furthermore, not-α IL2 molecules retain the capacity to induce signaling in Tregs (26–29), whose suppressive capacity is increased with IL2R activation (30). Collectively, these observations raise the possibility that the therapeutic index of IL2 could be improved by concentrating signaling on CD8^+^ T cells while avoiding other cell types such as NK cells and Tregs that may contribute to toxicity or oppose antitumor activity.

Here we report the design and preclinical evaluation of AB248, a CD8^+^ T cell–selective IL2. AB248 is a fusion of an attenuated IL2 mutein linked to an antibody targeting CD8β. AB248 achieves selective IL2R signaling on CD8αβ^+^ T cells via the principle of *cis*-targeting (31), in which preferential CD8^+^ T-cell signaling of an IL2 mutein is enabled by avidity provided by binding in *cis* to CD8β. AB248 recapitulates key features of IL2 biology, including induction of proliferation and enhancement of effector function, but does so selectively in CD8^+^ T cells across a wide range of concentrations *in vitro* and *in vivo*. The murine surrogate of AB248 (CD8-mIL2) demonstrates strongly improved antitumor activity and reduced toxicity compared with an untargeted not-α IL2, and effective therapy is characterized by increased numbers and enhanced function of tumorreactive CD8^+^ T cells. Collectively, these data provide proof of concept that the targeting of cytokines to specific immune cell subsets can improve the therapeutic index of pleiotropic cytokines and support the evaluation of AB248 for the treatment of cancer.

## Results

### CD8^+^ T Cells Drive Antitumor Activity of Not-α IL2

**Figure 1.**
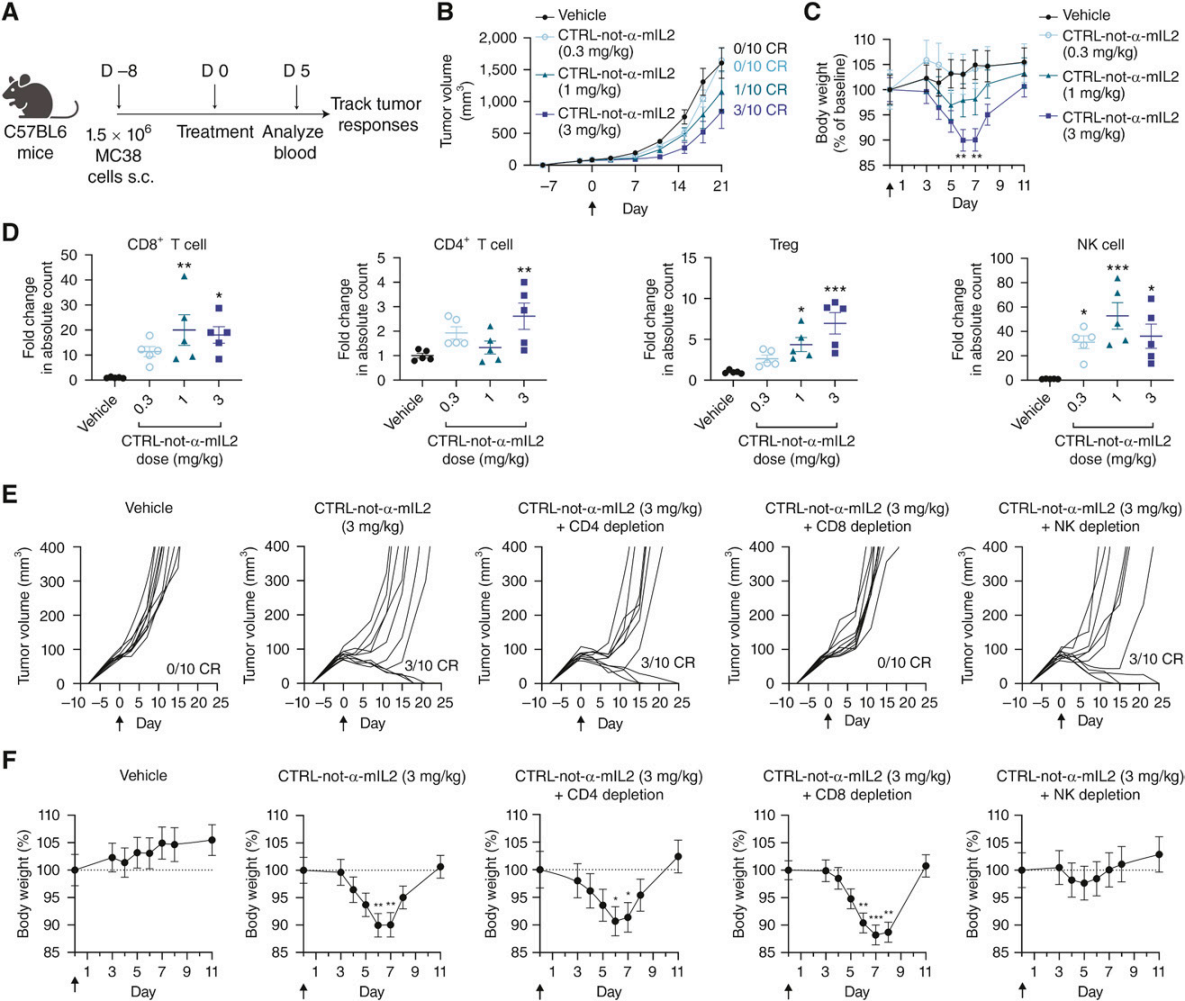
CD8^+^ T cells drive antitumor activity, but NK cells are responsible for toxicity with not-α-IL2 therapy. **A–F,** C57BL6 mice implanted with MC38 s.c. tumors were treated once with not-α IL2 (CTRL-not-α-mIL2) at the indicated doses. **A,** Schematic of the experimental design. Tumor size (**B**) and body weight (**C**) were assessed after treatment (*n* = 10). **D,** Peripheral blood cell expansion was assessed on day 5 after treatment with CTRL-not-α-mIL2, reported as fold change in absolute count over vehicle-treated mice (*n* = 5). **E** and **F,** Mice were depleted of CD4^+^ T cells, CD8^+^ T cells, or NK cells beginning 2 days prior to therapy and throughout therapy with CTRL-not-α-mIL2. Shown is tumor volume (**E**) and body weight (**F**; *n* = 10). Graphs in **B**, **C**, **D**, and **F** show mean ± SD. Studies are representative of 3–5 independent experiments. CR = complete response. Statistics were performed via one-way ANOVA with Dunnett multiple comparisons test versus vehicle (n.s., *P* > 0.05; *, *P* < 0.05; **, *P* < 0.01; ***, *P* < 0.001; ****, *P* < 0.0001).

To evaluate the extent to which antitumor activity and toxicity of IL2 can be uncoupled in mouse models, MC38 tumor-bearing mice were treated with a murine not-α IL2 expressed as a single fusion coupled to an IgG for half-life extension (CTRL- not-α-mIL2; Fig. 1A). Although partial antitumor activity was observed with increasing dose, body weight loss, a measure of toxicity, was observed at all doses at which tumor suppression occurred (Fig. 1B and C). In line with expectations, CTRL- not-α-mIL2 induced expansion of CD8^+^ T cells, CD4^+^ T cells, Tregs, and NK cells in blood, with NK cells demonstrating the greatest level of expansion (up to 50-fold; Fig. 1D). To evaluate the contribution of different cell types to antitumor activity and toxicity, CTRL-not-α-mIL2 therapy was administered to tumor-bearing mice that were depleted of either CD4^+^ cells (via anti-CD4), CD8^+^ cells (via anti-CD8β), or NK cells (via anti-NK1.1). Depletion of CD4^+^ T cells or NK cells did not hamper the antitumor activity of CTRL-not-α-mIL2, whereas CD8^+^ T-cell depletion abrogated antitumor activity (Fig. 1E). In contrast, whereas depletion of CD4^+^ or CD8^+^ T cells did not mitigate body weight loss, depletion of NK cells resulted in a substantial reduction in weight loss (Fig. 1F). Similar results were obtained with a not-α human IL2, which has attenuated binding to mouse IL2R (Supplementary Fig. S1A–S1C). Thus, NK cells contribute significantly to toxicity-induced body weight loss following therapy with not-α IL2 but are dispensable for antitumor activity, whereas CD8^+^ T cells are essential for antitumor activity but do not contribute to weight loss.

### Not-α IL2 Preferentially Activates Human NK Cells

**Figure 2.**
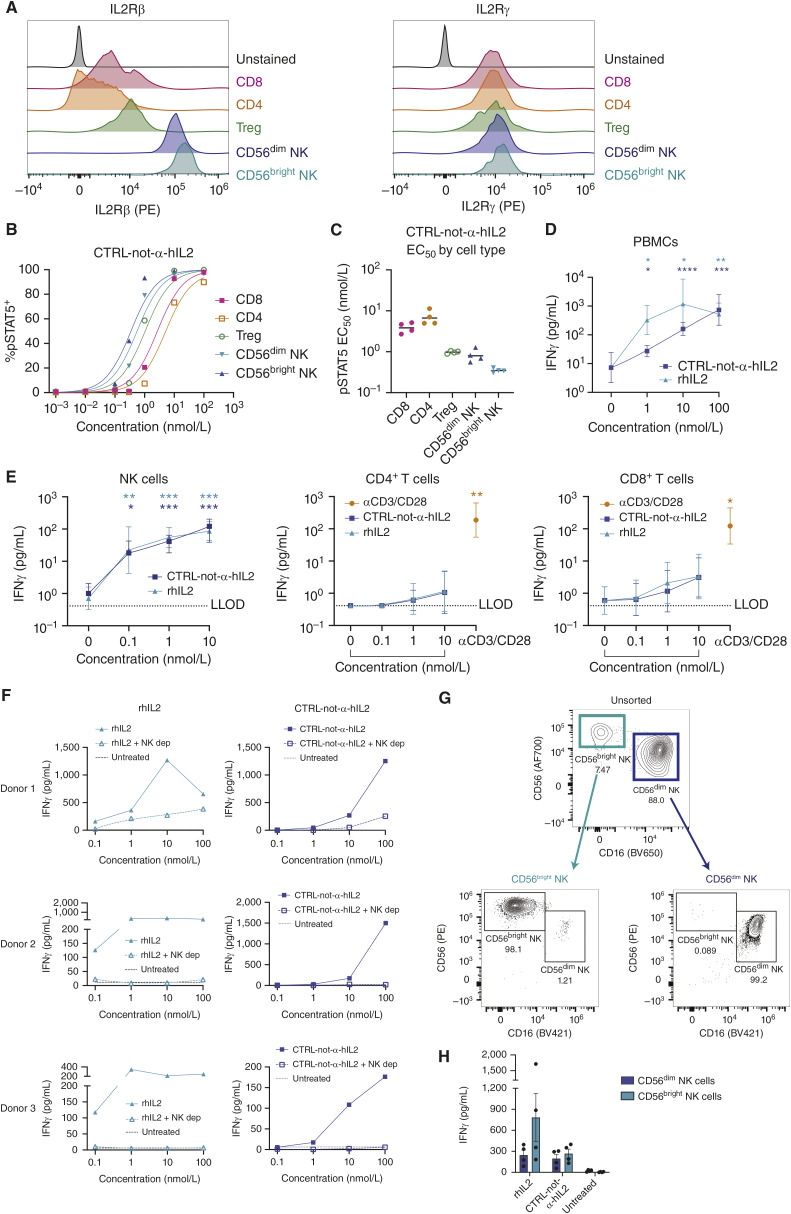
Human NK cells are preferentially activated by not-α-IL2 and exhibit antigen-independent IFNγ release *in vitro*. **A,** Human PBMC subsets were assessed for IL2Rβ or IL2Rγ expression by flow cytometry. **B** and **C,** pSTAT5 was assessed by flow cytometry following a 25-minute stimulation of human blood with CTRL-not-α-hIL2. Shown is the % pSTAT5^+^ within the indicated subsets as a function of CTRL-not-α-hIL2 concentration for a representative donor (**B**) and EC50 values from 4 donors for the indicated cell types (**C**). **D,** PBMCs were cultured for 24 hours in the presence of rhIL2 or CTRL-not-α-hIL2, and IFNγ was quantified in the supernatant using MSD (*n* = 10). **E,** NK cells, CD4^+^ T cells, or CD8^+^ T cells were isolated from human PBMCs via flow sorting and cultured with the indicated concentrations of rhIL2 or CTRL-not-α-hIL2 for 24 hours. TransAct CD3/CD28 stimulation (1:100) was used as a positive control for T-cell populations. Plotted are geometric mean ± geometric SD (*n* = 10). **F,** PBMCs or PBMCs depleted of NK cells via CD56 were cultured for 24 hours in the presence of rhIL2 or CTRL-not-α-hIL2, and IFNγ was quantified in the supernatant using MSD (*n* = 3). rhIL2; recombinant human IL2. **G** and **H,** Sorted CD56^bright^ and CD56^dim^ NK subsets were cultured as in **D** and **E**. Shown is representative sort strategy (**G**) and IFNγ in the supernatant (**H**; *n* = 4). Data shown in **B** and **C** are representative of more than 10 independent experiments; **A** and **D–H** are representative of 2–3 independent experiments. Statistics in **D** were performed via paired *t* test versus baseline values (n.s., *P* > 0.05; *, *P* < 0.05; **, *P* < 0.01; ***, *P* < 0.001; ****, *P* < 0.0001).

To evaluate IL2R expression levels in human immune cell populations, IL2Rβ and IL2Rγ were quantified on peripheral blood mononuclear cells (PBMCs). Human T-cell and NK cell populations showed similar levels of IL2Rγ, but IL2Rβ was expressed at substantially higher levels on both CD56^bright^ and CD56^dim^ NK cells as compared with T-cell populations (Fig. 2A). To assess sensitivity of different human immune cell types to IL2 signaling, a not-α IL2 (CTRL-not-α-hIL2) was assessed for pSTAT5 induction in human peripheral blood cells following The pSTAT5 EC_50_ values for NK cells (0.35 nmol/L for CD56^bright^ and 0.80 nmol/L for CD56^dim^) were 5- to 10-fold lower than for CD8^+^ T cells (3.83 nmol/L; Fig. 2B and C), in accordance with higher IL2Rβ levels on NK cells. Tregs exhibited ∼3× greater sensitivity (EC_50_ of 0.97 nmol/L) compared with CD8^+^ T cells (Fig. 2B and C). Hence, CTRL-not-α-hIL2 demonstrates an NK cell bias *in vitro* on human immune cells, and retains signaling activity on Tregs, in line with data on previously developed not-α IL2 variants (26–29).

### IL2 Signaling Induces Antigen-Independent Cytokine Release by NK Cells

Consistent with their role in innate immunity, NK cells have been reported to secrete proinflammatory cytokines such as IFNγ in response to IL2 signaling, unlike T-cell populations, which require a second signal such as T cell receptor (TCR) stimulation (32–34). Culture of human PBMCs with rhIL2 or CTRL-not-α-hIL2 resulted in dose-dependent secretion of IFNγ (Fig. 2D), and culture of sorted immune cell subsets from PBMCs revealed that NK cells robustly produced IFNγ in response to IL2 signaling, with elevations detectable at low concentrations (0.1 nmol/L) and to levels exceeding 100-fold over baseline (Fig. 2E). In contrast, T-cell populations produced only modest IFNγ at high concentrations which were not statistically elevated from baseline values. Depletion studies verified that IFNγ levels were substantially reduced upon depletion of NK cells from PBMCs (Fig. 2F). Furthermore, IFNγ secretion could be observed from both CD56^bright^ and CD56^dim^ NK cell populations (Fig. 2G and H). Thus, peripheral blood NK cells, but not T cells, exhibit antigen-independent IFNγ release in response to IL2 signaling.

### Evaluating Targeting Arms and IL2 Variants for CD8^+^ T-cell Targeting

**Figure 3.**
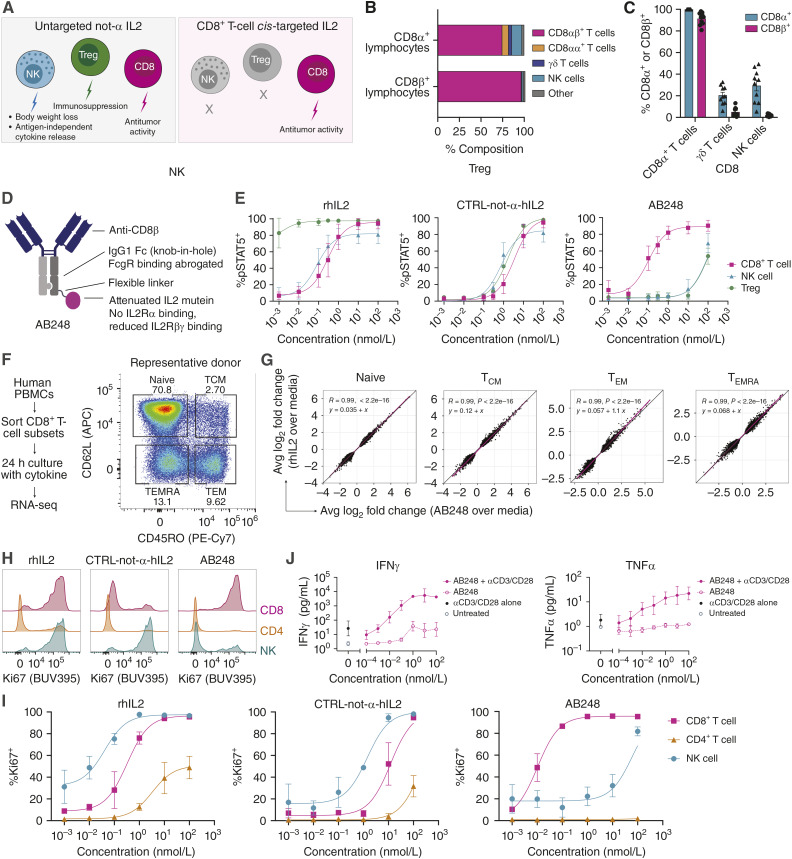
Molecular design and characterization of AB248, a CD8 *cis*-targeted IL2. **A,** Overview of the objectives for a CD8^+^ T-cell *cis*-targeted IL2 molecule. **B** and **C,** Human PBMCs were assessed for expression of CD8α and CD8β by flow cytometry (*n* = 10). Shown are the constituent cell types for CD8α^+^ or CD8β^+^ lymphocytes by percentage (**B**), and the percentage staining positive for CD8α or CD8β within the indicated cellular subsets (**C**). D, Overview of the molecular characteristics of the development candidate AB248. **E,** pSTAT5 was assessed by flow cytometry following a 25-minute stimulation of human blood with rhIL2, CTRL-not-α-hIL2, or AB248 (*n* = 10). **F** and **G,** Human CD8^+^ T-cell subsets were flow sorted from peripheral blood and cultured for 24 hours with AB248, rhIL2, or media, and transcriptional changes were analyzed via RNA-seq. Shown is the sorting strategy for a representative donor (**F**) and gene expression (by average log_2_ fold changes of cpm) over media control. APC; antigen presenting cell. (**G**) with each differentially expressed gene represented by a point. *P* value cutoffs of 0.2 are applied to fold change data. A reference dashed line with a slope of 1 is shown; R is the correlation coefficient from the linear regression model (*n* = 3 donors). **H** and **I,** human PBMCs were cultured with the indicated concentrations of rhIL2, CTRL-not-α-hIL2, or AB248 for 5 days and Ki-67 expression was assessed by flow cytometry. Shown is Ki-67 staining in one representative donor at 10 nmol/L (**H**) and Ki-67 expression (%) within the indicated cell subsets as a function of concentration (I; *n* = 2). **J,** Isolated peripheral blood CD8^+^ T cells were incubated with AB248 for 48 hours in the presence or absence of suboptimal TransAct CD3/CD28 stimulation (1:10,000) and supernatant IFNγ and TNFα were quantified using MSD (*n* = 5). Studies are representative of 2–3 independent experiments.

To address whether CD8^+^ T-cell selectivity improves the therapeutic index of IL2, we set out to develop a *cis*-targeted cytokine that efficiently signals on CD8^+^ T cells but not NK cells and Tregs (Fig. 3A). Analysis of CD8α and CD8β as potential target antigens showed that approximately 75% of human peripheral blood CD8α^+^ cells were conventional CD8αβ^+^ T cells, with the remaining 25% including CD8αα^+^ T cells, γδ T cells, and NK cells (Fig. 3B). Furthermore, roughly 20% of γδ T cells and 30% of NK cells showed expression of CD8α (Fig. 3C), and binding studies confirmed NK cell labeling by an anti-CD8α antibody (Supplementary Fig. S2A). In contrast, CD8β showed remarkable specificity for conventional CD8αβ^+^ T cells, with minimal expression by γδ T and NK cells (Fig. 3B and C). CD8β was thus chosen as a target antigen due to its restricted target expression on the cell type of interest.

The CD8αβ coreceptor plays an essential role in supporting TCR interactions with peptide:MHC during antigen encounter (35), and disruption of this coreceptor function is thus undesirable. We identified an antibody (clone 97/47) that is selective for CD8β over CD8α (Supplementary Fig. S2B), and that did not diminish CD8 coreceptor function as assessed by activation of CMV pp65-reactive CD8^+^ T cells in response to cognate peptide (Supplementary Fig. S2C).

To evaluate IL2 mutein properties that enable effective CD8^+^ T-cell targeting, while avoiding activation of nontarget cells at therapeutically relevant concentrations, fusions were generated between IL2 variants and either anti-CD8β (97/47) or an Fc control (Supplementary Table S1). Both Fc and anti-CD8β fusions to wild-type IL2 (wtIL2) or an IL2 mutein with reduced IL2Rβ binding (IL2αγ) retained the capacity to induce potent Treg activation (Supplementary Fig. S2D). In contrast, anti-CD8β fusions to not-α IL2 (CD8-IL2βγ), or to a variant with no IL2Rα binding and reduced IL2Rβ binding (CD8-IL2β^*^γ), demonstrated >500-fold preference for CD8^+^ T cells. In human PBMC cultures, both elimination of IL2Rα binding and reduction of IL2Rβ binding were necessary to avoid antigen-independent IFNγ release, regardless of whether the mutein was targeted to CD8β (Supplementary Fig. S2E). Therefore, to avoid both Treg activation and antigen-independent cytokine release, the IL2β^*^γ mutein with no IL2Rα binding and reduced IL2Rβ binding was selected. Fusion of IL2β^*^γ to a CD8α-targeting antibody demonstrated both more potent NK cell activation and substantially greater antigen-independent cytokine release as compared with that observed with a CD8β-targeting antibody (Supplementary Fig. S2F and S2G), confirming CD8β as the more suitable target. We proceeded with the characterization of the development candidate CD8β-IL2β^*^γ, termed AB248 (Fig. 3D).

### *In Vitro* Characterization of AB248, a Human CD8-IL2

AB248 demonstrated an EC_50_ of 0.10 nmol/L for pSTAT5 induction on human CD8^+^ T cells, over 500-fold greater sensitivity than was observed for other cells (59.1 nmol/L for NK cells; >100 nmol/L for Tregs), whereas rhIL2 and CTRL-not-α-hIL2 activated CD8^+^ T cells with similar or lower potency than NK and Tregs (Fig. 3E). Evaluation of transcriptional changes following stimulation of naive, central memory (T_CM_), effector memory (T_EM_), and effector memory reexpressing CD45RA (T_EMRA_) CD8^+^ T-cell subsets confirmed that AB248 recapitulated the signature induced by rhIL2 with high fidelity (Fig. 3F and G). To assess proliferation, Ki-67 was measured after 5 days of culture. Although rhIL2 and CTRL-not-α-hIL2 exhibited 8.0- and 8.6-fold more potent Ki-67 induction in NK cells compared with CD8^+^ T cells, AB248 showed >6,000-fold more potent Ki-67 induction in CD8^+^ T cells compared with NK cells (Fig. 3H and I). Cytokine release assays confirmed that AB248 induced minimal release of proinflammatory cytokines from human PBMCs in contrast to both rhIL2 and CTRL-not-α-hIL2, which induced dose-dependent release of IFNγ, TNFα, IL5 and IL6 (Supplementary Fig. S2H). Furthermore, AB248 exhibited a high degree of stability, with no evidence of degradation or cleavage after incubation in human serum at 37°C for one week (Supplementary Fig. S2I and S2J). Although signaling via IL2R alone is insufficient to promote effector cytokine secretion from CD8^+^ T cells, IL2 signaling is known to enhance effector cytokine secretion by antigen-activated CD8^+^ T cells (16, 34). In line with these data, CD8^+^ T cells receiving TCR stimulation showed increased levels of IFNγ and TNFα secretion in the presence of AB248 (Fig. 3J). Thus, AB248 recapitulates IL2 signaling and function *in vitro* but does so with CD8^+^ T-cell selectivity.

### CD8 Cis-Targeted IL2 Therapy Drives Potent Antitumor Activity

**Figure 4.**
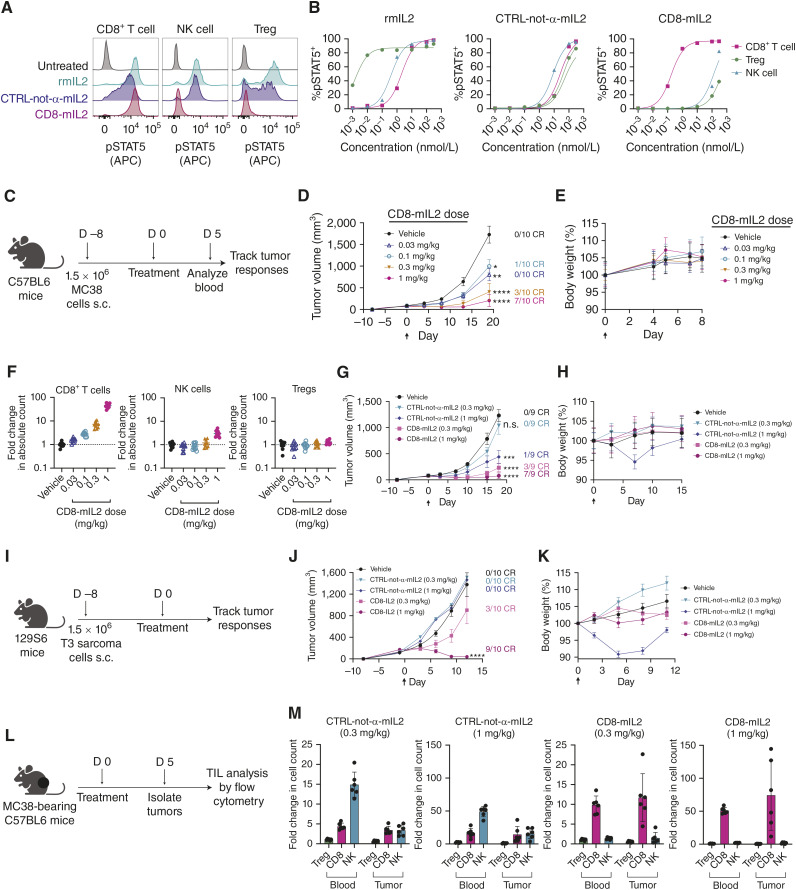
Therapy with CD8 *cis*-targeted IL2 in mice results in potent antitumor activity. **A** and **B,** Mouse splenocytes were stimulated for 25 minutes with the indicated molecules and pSTAT5 was assessed by flow cytometry. Shown is representative pSTAT5 staining at 10 nmol/L (**A**) and frequency of pSTAT5^+^ for the indicated cell types as a function of concentration (**B**). C–H, C57BL6 mice were implanted with MC38 tumors s.c. and treated i.v. 8 days later with indicated therapy. Shown is the study schema (**C**), and tumor volume (**D**), and body weight (**E**) over time following dosing with CD8-mIL2. **F,** Peripheral blood was analyzed 5 days after CD8-mIL2 dosing by flow cytometry; shown is fold change in absolute cell count over vehicle-treated animals for the indicated cell types (*n* = 10). **G** and **H,** Mice were implanted with MC38 tumors and treated 8 days later once with CD8-mIL2 or CTRL-not-α-mIL2, and tumor size. (**G**) and body weight (**H**) were assessed (*n* = 9). **I–K,** 129S6 mice were implanted with T3 sarcoma cells and treated 8 days later once with the indicated treatments. Shown are the study schema (**I**), tumor volume (**J**), and body weight (**K**; *n* = 10). L and M, MC38-bearing mice were treated once with CD8-mIL2 or CTRL-not-α-mIL2 and absolute cell counts were quantified from peripheral blood and tumors 5 days after treatment. Cell counts are plotted as fold change over vehicle-treated animals (*n* = 6). Data represented as mean ± SD, and data shown are representative of 2–4 independent experiments. Statistics in **D**, **G**, and **J** were performed via one-way ANOVA with Dunnett multiple comparisons test versus control at the latest time point plotted (n.s., *P* > 0.05; *, *P* < 0.05; **, *P* < 0.01; ***, *P* < 0.001; ****, *P* < 0.0001). CR = complete response.

To evaluate the therapeutic potential of CD8^+^ T cell-targeted IL2, a potency-matched CD8-mIL2 ( Fig. 4A and B). Evaluation of the biodistribution of intravenously administered CD8-mIL2 in mice demonstrated highly preferential labeling of CD8^+^ T cells by CD8-mIL2 in all tissues evaluated, including in subcutaneously implanted tumor (Supplementary Fig. S3A–S3C). Conversely, CTRL-not-α-mIL2 could be detected broadly in IL2R^+^ cell types but showed preferential labeling of NK cells. MC38-bearing mice treated with a single dose of CD8-mIL2 at day 8 demonstrated dose-dependent antitumor activity, with most mice (7/10) achieving durable tumor rejection at 1 mg/kg (Fig. 4C and D). Notably, mice that responded to CD8-mIL2 therapy rejected rechallenge with MC38, indicating the induction of immunologic memory (Supplementary Fig. S4A and S4B). Tumor regression occurred without body weight loss (Fig. 4E) and was accompanied by dose-dependent, selective CD8^+^ T-cell expansion in blood (Fig. 4F). Concurrent therapy with the S1P1R agonist FTY720, which blocks lymphocyte egress from lymph nodes, demonstrated a partial reduction in antitumor activity, suggesting that both preexisting tumor-resident and newly infiltrating lymphocytes contribute to activity (Supplementary Fig. S4C and S4D). CD8-mIL2 outperformed CTRL-not-α-mIL2 in both the MC38 and the 3-methylcholanthrene (MCA) induced d42m1-T3 sarcoma (T3) models (Fig. 4G–K), demonstrating both superior antitumor activity and reduced body weight loss. A single dose of CD8-mIL2 also outperformed daily dosing of IL2 (100,000 IU/day; Supplementary Fig. S4E–S4G). To characterize changes in immune cell composition after therapy, cellular expansion was quantified in blood and tumors (Fig. 4L): CTRL-not-α-mIL2 induced a strong expansion of peripheral blood NK cells, with more modest changes to intratumoral populations, whereas CD8-mIL2 demonstrated robust and selective CD8^+^ T-cell expansion, both in blood and at the tumor site (Fig. 4M). Interestingly, the magnitude of intratumoral CD8^+^ T-cell expansion induced by CD8-mIL2 was comparable with that observed for peripheral blood CD8^+^ T cells, despite the expected lower drug exposure in tumor compared with the periphery. This observation may be consistent with a lower threshold for proliferation induction for CD8^+^ tumor-infiltrating lymphocytes (TIL), which may be receiving a concurrent antigen signal, or may signify migration of cells from the periphery into the tumor following therapy. To evaluate the potential of CD8 *cis*-targeted IL2 in the context of adoptive cell therapy, A375 melanoma-bearing NSG mice were treated with human CAR-T cells specific for B7H3 with or without CD8-hIL2 (Supplementary Fig. S4H). In this model, CAR-T cells alone resulted in only a minor tumor growth delay, but the addition of CD8-hIL2 resulted in complete rejection of tumor in all mice treated (Supplementary Fig. S4I). Thus, CD8 *cis*-targeted IL2 shows robust activity as monotherapy in multiple models and may additionally have the potential to augment adoptive cell therapy.

### CD8-mIL2 in Combination with Anti–PD-1 Is Effective in Anti–PD-1-Resistant Tumor Models

**Figure 5.**
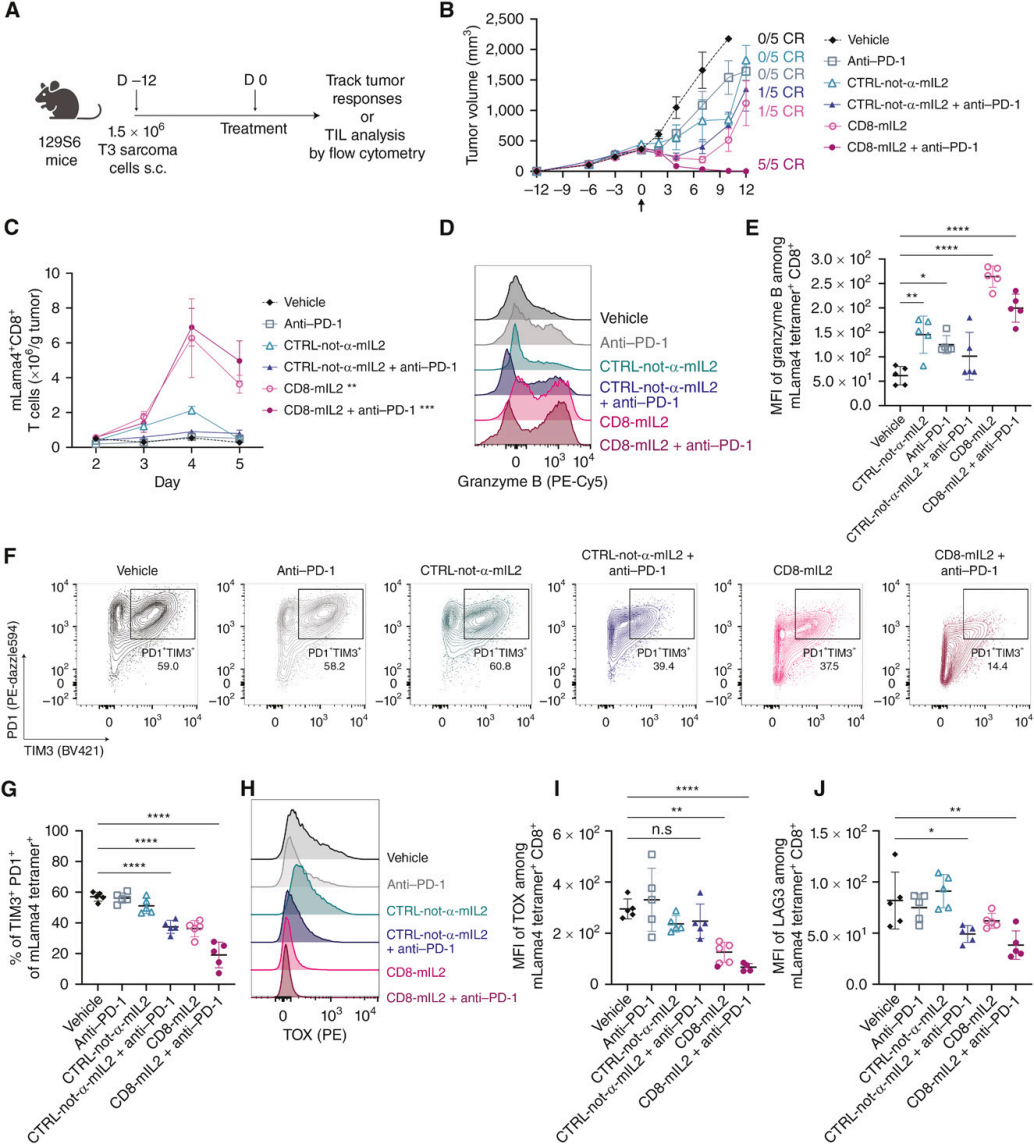
CD8 *cis-*targeted IL2 enhances the number and function of tumor antigen–reactive CD8^+^ T cells in mice. **A** and **B,** 129S6 mice were implanted with T3 sarcoma cells and treated 12 days later with a single dose of 1 mg/kg of CTRL-not-α-mIL2 or CD8-mIL2 as monotherapy or in combination with 5 mg/kg of anti–PD-1. Shown are the study schema (**A**) and tumor volume after therapy (**B**; *n* = 5). **C–J,** T3-bearing mice were treated with the indicated therapy and TILs were isolated and stained with mLama4 tetramer and analyzed by flow cytometry following therapy. **C,** Absolute counts of mLama4 tetramer^+^ CD8^+^ T cells in the tumor were assessed over time. **D** and **E,** Granzyme B expression in mLama4 tetramer^+^ TILs 2 days after therapy. Shown is granzyme B staining from representative mice (**D**) and across all mice (**E**; *n* = 5). **F–J,** Expression of exhaustion-associated markers by mLama4 tetramer^+^CD8^+^ TILs was assessed by flow cytometry 5 days following therapy. Shown are representative staining among mLama4 tetramer^+^ CD8^+^ T cells (**F**), the % TIM3^+^PD-1^+^ of mLama4 tetramer^+^ CD8^+^ T cells (**G**), TOX staining of mLama4 tetramer^+^ CD8^+^ T cells in representative mice (**H**) and across all mice (**I**), and expression of LAG3 within mLama4 tetramer^+^ CD8^+^ T cells (J: *n* = 5). Data, mean ± SD; data are representative of 2 independent experiments. Statistical analyses were performed via one-way ANOVA with Dunnett multiple comparisons test (n.s., *P* > 0.05; *, *P* < 0.05; **, *P* < 0.01; ***, *P* < 0.001; ****, *P* < 0.0001). Statistics in **C** were calculated on day 4. CR = complete response.

Preclinical and clinical evidence supports that IL2 pathway activation along with PD-1 checkpoint blockade is a promising immunotherapy strategy (4, 36, 37). To evaluate the combination potential of CD8-mIL2 and anti–PD-1, T3 tumor-bearing mice were treated on day 12, at which time tumors became resistant to anti–PD-1 therapy (Fig. 5A; ref. 38). CTRL-not-α-mIL2 or anti–PD-1 monotherapy showed minimal antitumor activity, and the combination of the two showed only a modest improvement in activity (Fig. 5B). CD8-mIL2 monotherapy induced tumor regression transiently, although tumors in most mice ultimately grew out. However, in combination with anti–PD-1, striking therapeutic benefit was observed, with all mice demonstrating durable complete tumor rejection. Strong anti–PD-1 combination benefit was observed in four additional settings: B16F10, 1956 sarcoma, MCA205, and KP.mLama4 (38) tumor models (Supplementary Fig. S5A–S5I), all of which showed limited activity with anti–PD-1 monotherapy in the treatment settings used.

### Antigen-Specific CD8^+^ T Cells Increase in Number and Function after CD8-mIL2 Therapy

To assess how CD8-mIL2 treatment influences tumor antigen–reactive CD8^+^ T-cell responses at the tumor site, T3 sarcoma tumors were analyzed using MHC tetramers loaded with the immunodominant H-2K^b^-restricted mLama4 antigen (39). Counts of intratumoral mLama4-reactive CD8^+^ T cells were substantially increased with CD8-mIL2 treatment, both upon monotherapy and upon combination treatment with anti–PD-1, whereas CTRL-not-α-mIL2 treatment showed only a minor increase in mLama4-reactive cells (Fig. 5C). Granzyme B expression in mLama4-reactive cells was only slightly increased after therapy with anti–PD-1, CTRL-not-α-mIL2, or the combination of the two, but was substantially enhanced by CD8-mIL2 and CD8-mIL2 + anti–PD-1 therapy (Fig. 5D and E). Notably, levels of exhaustion-associated markers PD-1, TIM3, LAG3, and TOX by intratumoral mLama4-reactive cells were dramatically reduced in CD8-mIL2 + anti–PD-1-treated mice and modestly affected in other groups (Fig. 5F–J). Analysis of TILs from MC38 tumors with MHC tetramers loaded with the H-2K^b^-restricted neoantigen Rpl18 (40) likewise showed increased numbers of tumor antigen–reactive CD8^+^ T cells and decreased expression of PD-1 following therapy with CD8-mIL2 (Supplementary Fig. S6A–S6E). Antigen-reactive T_CM_, T_EFF_, and T_EM_ cell numbers were all increased in CD8-mIL2–treated mice without a significant change in proportional makeup (Supplementary Fig. S6F–S6H). Therefore, compared with CTRL-not-α-mIL2, CD8^+^ T-cell *cis*-targeted IL2 shows a superior ability to increase the numbers and functionality of intratumoral antigen-specific CD8^+^ T cells in multiple tumor models.

### Phenotypes of Tumor-Infiltrating CD8^+^ T Cells Are Markedly Enhanced Following CD8-mIL2 Therapy

**Figure 6.**
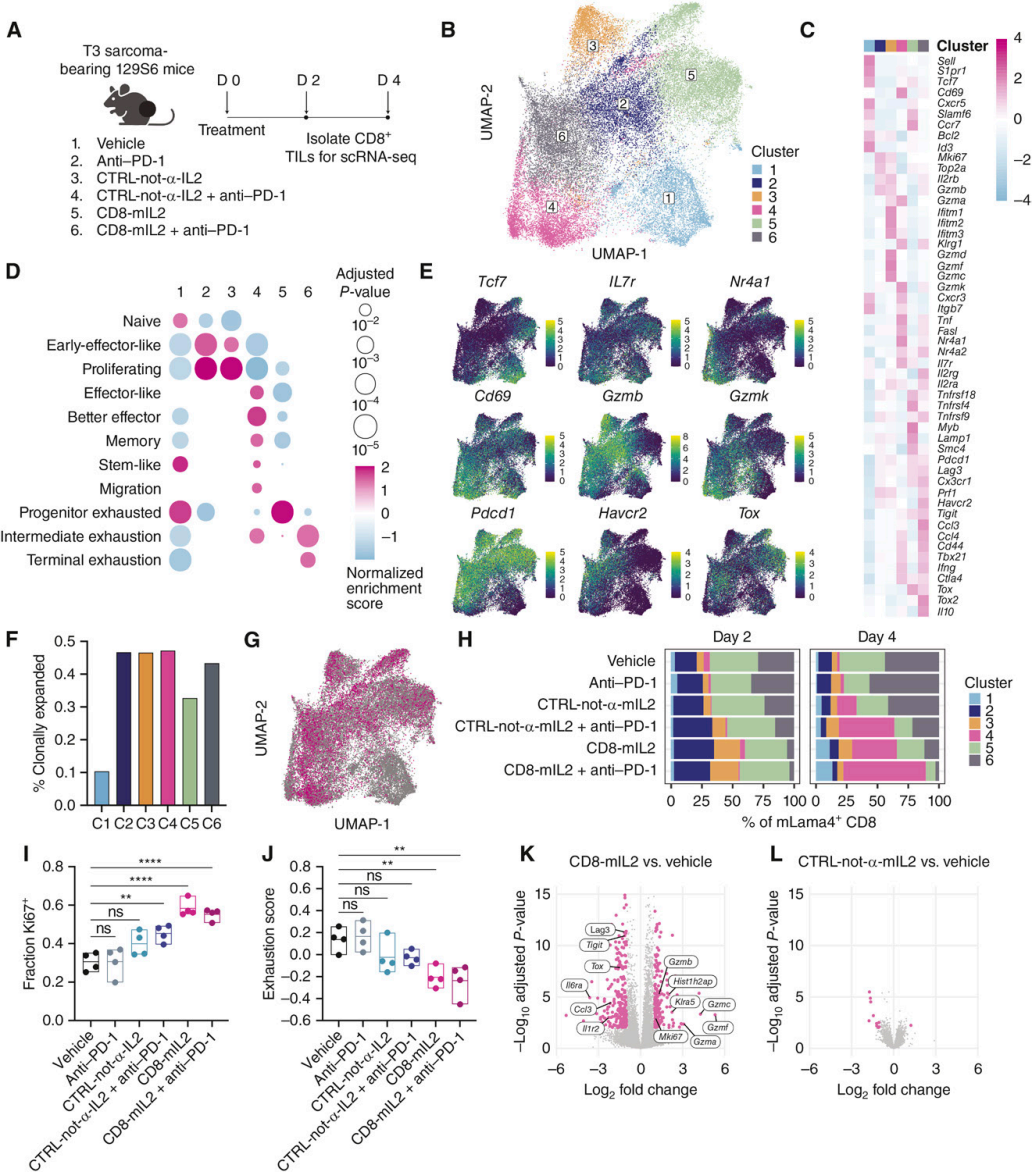
scRNA-seq after CD8-mIL2 therapy reveals dramatic rewiring of CD8^+^ T-cell immunity. **A–L,** 129S6 mice were implanted with d42m1-T3 (T3) sarcoma cells and treated 12 days later with a single dose of 1 mg/kg of CTRL-not-α-mIL2 or CD8-mIL2 as monotherapy or in combination with 5 mg/kg of anti–PD-1. Tumors were isolated 2 and 4 days after therapy and CD8^+^ T cells were flow sorted and scRNA-seq (10× Genomics) was performed with BEAM reagent labeling of mLama4-specific CD8^+^ T cells (*n* = 4 per group per day). **B,** UMAP visualization of CD8^+^ TILs according to the cluster. **C,** Relative expression (z-score of cluster average log-normalized counts) of selected genes across CD8^+^ T-cell clusters. **D,** Gene-set enrichment analysis of differentially expressed genes per identified CD8^+^ T-cell cluster. **E,** Expression profile of selected genes. **F,** Percentage clonally expanded, defined as detection of 3 or more cells with a given TCR sequence, by cluster. **G,** BEAM-labeled identification of mLama4-reactive CD8^+^ T cells. **H,** Proportion of mLama4-specific CD8^+^ TILs in each cluster on day 2 and day 4 by treatment condition. Colors correspond to the clusters as in **B** and **F**. **I,** Ki-67 expression by treatment condition in mLama4-specific CD8^+^ TILs. **J,** Exhaustion signature scores among mLama4-specific CD8^+^ TILs by treatment group. **K** and **L,** Volcano plots for differentially expressed genes versus vehicle control in mLama4-reactive CD8^+^ TILs for CD8-mIL2 (**K**) and CTRL-not-α-mIL2 (**L**).

To assess treatment-induced changes of intratumoral CD8^+^ T cells in an unbiased manner, CD8^+^ TILs from T3 sarcomas were analyzed using single-cell RNA-sequencing (scRNA-seq) 2 or 4 days after therapy with CD8-mIL2, CTRL-not-α-mIL2, anti–PD-1, or combinations thereof (Fig. 6A). Barcoded peptide:MHC reagents were included to identify mLama4-specific CD8^+^ T cells. The analysis yielded single-cell transcriptomes from 39,406 TIL CD8^+^ T cells, of which 11,879 were identified as mLama4-reactive. Unbiased clustering of transcriptomic signatures identified six subsets (C1–C6) of CD8^+^ TILs based on the expression of known marker gensses and previously defined gene-expression signatures (Fig. 6B–E; refs. 41–44). These CD8^+^ T-cell clusters included a naive-like/recently activated cluster C1 (*Sell*^+^, *S1pr1*^+^, *Cd69*^+/−^; Supplementary Fig. S7A–S7D), two clusters of proliferating effector CD8^+^ TILs, C2 (*Gzmb*^+^, *Prf1*^+^, *Ki-67*^+^) and C3 (*Ifitm1*^+^*Gzmc*^+^*Gzmf*^+^, *Ki-67*^+^), a population with characteristics of both memory and effector function C4 (*Ifng*^+^, *Tnfa*^+^, *Gzma*^+^, *Il7r*^+^), and two clusters of *Pdcd1*^+^ exhausted cells: precursor exhausted C5 (*Tcf7*^+^, *Ccr7*^+^, *Slamf6*^+^) and terminally exhausted C6 (*Tox2*^+^*, Havcr2*^+^, *IL10*^+^). In accordance, C2–C6 exhibited clonal expansion and were enriched for mLama4-reactive CD8^+^ T cells (Fig. 6F and G; Supplementary Fig. S7E–S7G).

In vehicle and anti–PD-1–treated animals, the majority (>60%) of mLama4-reactive CD8^+^ T cells fell into exhaustion-associated C5 and C6, whereas effector populations C2, C3, and C4, comprised less than 25% of cells at both time points tested (Fig. 6H; Supplementary Fig. S7H). In contrast, on day 2, CD8-mIL2 monotherapy and CD8-mIL2 + anti–PD-1 combination therapy induced a pronounced increase in the two proliferating (Ki-67^+^) effector clusters C2 and C3 and a substantial reduction in terminally exhausted C6 to less than 6% of CD8^+^ T cells, compared with 30% in controls. At the 1 mg/kg dose of CTRL-not-α-mIL2, a dose at which toxicity is already observed (see Fig. 4H and K), more modest but directionally similar changes in CD8^+^ TIL subsets were observed in mice treated with or without anti–PD-1. On day 4, a striking treatment-induced effector population C4 emerged within tumor-reactive TILs, most prominently in the CD8-mIL2 + anti–PD-1 group (>66%), while being nearly absent in vehicle-treated animals (<3%). This C4 population was characterized by the expression of cytokines (*Ifng*, *Tnfa*), effector molecules (*Fasl*, *Gzma*), and *Il7r*, suggesting that C4 is an activated effector population with characteristics of a recently described “better effector” population (42). C4 was also increased to a lesser extent in CD8-mIL2 monotherapy and CTRL-not-α-mIL2 + anti–PD-1–treated mice, and was modestly increased upon CTRL-not-α-mIL2 monotherapy. Analysis of pan-CD8^+^ T cells revealed generally similar trends in treatment-induced cluster distribution changes, with a slightly higher frequency of C1 across conditions (Supplementary Fig. S7I). Both CD8-mIL2 monotherapy and CD8-mIL2 + anti–PD-1 combination therapy enhanced proliferation scores and decreased the exhaustion score of mLama4-reactive CD8^+^ T cells (Fig. 6I and J; Supplementary Fig. S8A). Comparing differentially expressed genes among mLama4^+^ CD8^+^ T cells revealed a markedly altered transcriptional program two days after CD8-mIL2 treatment, whereas effects with CTRL-not-α-mIL2 were modest (Fig. 6K and L; Supplementary Fig. S8B and S8C).

Analysis of CD8-mIL2-treated MC38 tumors 3 days after therapy showed similar trends in the CD8^+^ T-cell compartment: notable increases in effector populations, including substantial increases in a cluster with features of “better effectors,” C5 (Supplementary Fig. S9A–S9E). In addition, although myeloid population frequencies were not substantially altered at this time point, a strong induction of the IFNγ response signature could be detected across all myeloid clusters (Supplementary Fig. S9F–S9J), which may be consistent with augmented IFNγ production by tumor antigen–reactive CD8^+^ TILs following CD8-mIL2 therapy.

The finding that CD8-mIL2 therapy promotes the generation of a new effector CD8^+^ T-cell cluster with a signature indicative of polyfunctionality and with features of T-cell memory supports the notion that CD8^+^ T cell–selective IL2 promotes effective, potent, and durable antitumor immunity, which may be further augmented by anti–PD-1 combination therapy in challenging therapeutic settings.

### AB248 Selectively Expands CD8^+^ T Cells in Primates

**Figure 7.**
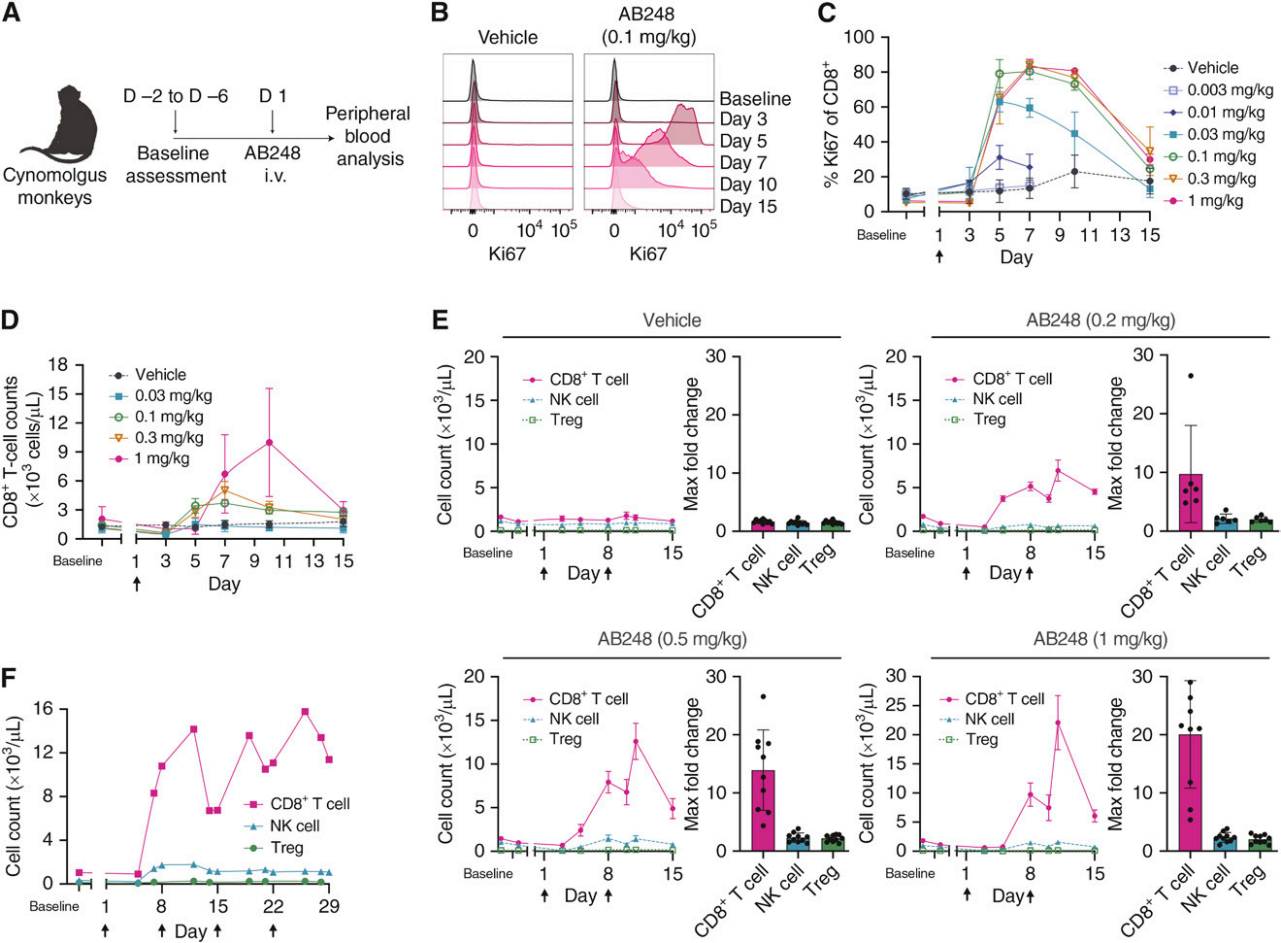
AB248 selectively expands CD8^+^ T cells in nonhuman primates. **A–F,** Cynomolgus monkeys were dosed i.v. with AB248 and peripheral blood was analyzed by flow cytometry. Shown are the study schema (**A**), representative Ki-67 staining in CD8^+^ T cells (**B**), and the percentage of peripheral blood CD8^+^ T cells staining positive for Ki-67 over time for the indicated AB248 doses (**C**; *n* = 2–4 per dose level). Absolute cell quantitation was performed using flow cytometry and hematology on the same blood draw. Shown are absolute CD8^+^ T-cell counts at the indicated dose levels following a single AB248 dose (**D**, *n* = 2–4 per dose level) and absolute counts and fold change of the indicated cell types following two AB248 doses (**E**, *n* = 6–10 per dose level). **F,** A cynomolgus monkey was dosed intravenously weekly with AB248 at 0.5 mg/kg for four doses and the indicated immune cell counts were assessed via flow cytometry and hematology. Doses of AB248 are indicated with arrows. Data in **C**, **D**, and **E,** mean ± SD.

AB248 showed comparable CD8^+^ T-cell binding and pSTAT5 potency on cynomolgus monkey cells compared with human cells (Supplementary Fig. S10A and S10B), confirming cynomolgus monkeys are suitable for evaluation of AB248’s *in vivo* activity. In cynomolgus monkeys dosed intravenously with AB248, dose-dependent induction of Ki-67 in peripheral blood CD8^+^ T cells was observed at doses ≥0.01 mg/kg (Fig. 7A–C). At doses that induced sustained Ki-67 expression (≥0.1 mg/kg), dose-dependent CD8^+^ T-cell expansion was observed (Fig. 7D; Supplementary Fig. S10C–S10G). With two weekly doses, AB248 demonstrated expansion of CD8^+^ T cells up to 20-fold, with minimal changes to NK cell, CD4^+^ T cell, and Treg cell counts (within 2.2-fold of starting counts at all dose levels; Fig. 7E). Furthermore, the magnitude of CD8^+^ T-cell expansion was comparable to that achieved with CD8-mIL2 at efficacious dose ranges in mouse tumor studies (Fig. 4F). CD8^+^ T cells could be repeatedly reexpanded, without diminution of peak counts or accumulation of nontargeted cell types for at least 4 weekly doses (Fig. 7F). AB248 was generally well tolerated at doses tested up to 1 mg/kg weekly, with no fever, hypotension, eosinophilia, or adverse clinical pathology or histopathology findings at any dose level. At 1 mg/kg, transient diarrhea occurred in some animals and resolved without treatment. Thus, *cis*-targeting of IL2 to CD8^+^ T cells enables safe, potent, and selective CD8^+^ T cell expansion in nonhuman primates at dose levels corresponding to highly efficacious mouse dose levels.

## Discussion

In view of the evidence supporting a key role for CD8^+^ T cells in IL2-induced efficacy, we set out to design a molecule capable of reproducing native IL2 signaling selectively in CD8^+^ T cells. Given their established role in tumor recognition, we elected to focus on conventional CD8αβ^+^ T cells (45). We verified that AB248, a CD8αβ^+^ T-cell *cis*-targeted IL2 mutein, induces native IL2 signaling (Fig. 3F and G) and that key functions such as the induction of proliferation and enhancement of effector function are preserved (Fig. 3H–J). The AB248 mouse surrogate (CD8-mIL2) demonstrated robust activity in several models (Fig. 4D, G, and J), and therapy was associated with increased numbers and functions of tumor antigen–reactive CD8^+^ TILs (Fig. 6C–J), including the emergence of a population characterized by a “better effector” signature (Fig. 6D and H; ref. 42). Combination with anti–PD-1 appeared to further increase the size of the “better effector” population in CD8^+^ TILs (Fig. 6H), which was associated with complete tumor rejection in advanced T3 sarcomas in mice. In human tumors, data in the accompanying article (Kaptein and colleagues) confirm the ability of AB248 to reinvigorate intratumoral CD8^+^ T cells to elicit productive antitumor immunity, including in tumors that showed no immunologic response to anti–PD-1 treatment. Collectively, these data support the therapeutic potential of CD8^+^ T cell–selective IL2 for the treatment of cancer.

Recent reports suggest that the emergence of a “better effector” intratumoral CD8^+^ T-cell population after treatment with IL2 correlates with strong antitumor activity in mice (42, 46). Notably, these cells showed reduced expression of the transcription factor *Tox*, which has a fundamental role in driving terminal exhaustion of CD8^+^ T cells (47). Indeed, in tumor antigen–reactive TILs in mice, CD8^+^*cis*-targeted IL2 showed a marked ability to markedly diminish *Tox* expression (Fig. 5I) and reduce features of exhaustion (Fig. 6J), findings that were also seen in the accompanying manuscript in human TILs (Kaptein and colleagues). A recent publication detailed a role for STAT5, the major downstream signal of the IL2 receptor, in antagonizing *Tox* expression and constraining the ability of CD8^+^ T cells to transition to terminal exhaustion (48). Authors demonstrate that constitutive STAT5 activation induces a marked rewiring of the epigenetic landscape of CD8^+^ T cells, with greater accessibility of effector-related peaks and decreased accessibility of exhaustion-related peaks. Following constitutive STAT5 activation, these epigenetically reprogrammed CD8^+^ T cells showed higher expression of gene modules associated with cytokine production, cytotoxicity, and activation, including genes such as *Gzma*, *Gzmb*, *Klrg1*, and *Tbx21*, along with decreased expression of an exhaustion module, including reduction in *Tox*, *Pdcd1*, and *Lag3*. Notably, these changes are also observed in tumor antigen–reactive CD8^+^ T cells following CD8-mIL2 therapy (Figs. 5D–J and 6C, H, J, K). Future work analyzing epigenetic changes after therapy may shed light on whether similar epigenetic rewiring is observed in response to exogenous CD8^+^*cis*-targeted IL2 therapy.

Numerous not-α IL2 variants have demonstrated an NK cell bias in their pharmacologic effects in patients (21–24), and these IL2Rβγ agonists show a similar NK cell bias *in vitro* (Figs. 2B, C and 4A, B) and *in vivo* in mice (Fig. 1D). Depletion studies in mice implicate NK cells in the body weight loss that is observed with not-α IL2 treatment, whereas CD8^+^ T cells were responsible for efficacy (Fig. 1E and F). This set of observations may explain why body weight loss and anti-tumor activity could not be decoupled with not-α IL2 therapy (Fig. 1B and C). In human PBMCs, NK cells but not CD8^+^ T cells exhibited antigen-independent IFNγ release (Fig. 2D and E), which may pose a toxicity risk in patients (49). In aggregate, these data suggest that the intrinsic NK cell bias exhibited by untargeted IL2 variants is undesirable.

The role of CD4^+^ T cells in antitumor immunity has been extensively documented (50), but CD4^+^ T cells were dispensable for activity with not-α IL2 therapy (Fig. 1E), consistent with data using other IL2-based therapies (14, 15, 17, 51). An important function of CD4^+^ T cells is to provide cytokine support to CD8^+^ T cells, so it is conceivable that exogenous IL2 may compensate for an absence of CD4^+^ T cell–derived IL2. Alternatively, this apparent CD4^+^ T-cell dispensability may arise from the opposing effects of IL2 signaling on both effector CD4^+^ T cells and Tregs.

Besides IL2, several engineered variants of IL15, which also signals via IL2Rβγ, have been developed (52). In clinical studies, these IL15-based IL2Rβγ agonists also show a pharmacodynamic bias toward NK cell activation over CD8^+^ T-cell activation, and collectively have shown little single-agent clinical activity in patients (53–55). In line with the data presented here, toxicity from IL15-based IL2Rβγ agonists depends on NK cells, whereas antitumor activity depends on CD8^+^ T cells in mice (13, 56, 57). Thus, the *in vivo* behavior of untargeted IL2- and IL15-based IL2Rβγ agonists appears largely comparable, which is consistent with similar downstream signaling induced *in vitro* by IL2 and IL15 (58).

Recent reports have suggested a role of IL2Rα engagement for optimal antitumor or antiviral immunity (36, 59); however, our data reveal potent monotherapy activity with CD8-mIL2 without IL2Rα engagement. Because IL2Rα has no signaling-competent intracellular domain (60), it predominantly acts as a modifier to increase the sensitivity to IL2 signaling on expressing cell types, a feature that can be recapitulated by *cis*-targeting. A recent study (42) demonstrated that PD-1 *cis*-targeted IL2 may obviate the need for IL2Rα engagement to generate robust antitumor activity, and our data suggest that this is also enabled via CD8β targeting. It is noted that the elimination of IL2Rα binding was essential to avoid Treg activation (Fig. 3E) and may also be important for avoiding VLS-associated toxicities and eosinophilia (61–63), providing a further rationale for this design.

Alternative approaches aimed at biasing IL2’s activity toward cytokine receptor subunits have been proposed (64, 65). However, selectivity thresholds achievable with these strategies are likely to be bounded by receptor expression levels on each cell type, and thus may not yield the selectivity window of ∼1,000-fold or greater that is achievable by *cis*-targeting. Efforts have also been made to localize IL2 activity to tumors as a strategy for improving IL2’s therapeutic index, for example using protease-sensitive masking (66, 67). It remains to be seen whether such approaches will be able to overcome interpatient and intratumoral variability in protease expression and achieve sufficient discrimination between tumor and healthy tissue to widen IL2’s therapeutic index in patients. Notably, systemic administration of CD8-mIL2 was not accompanied by overt toxicities at efficacious doses in mice or the corresponding AB248 doses in primates. This may be consistent with the observation that, as adaptive lymphocytes, CD8^+^ T cells require a second signal such as antigen recognition to secrete effector cytokines (Figs. 2E and 3J; ref. 34). Furthermore, the fact that AB248-induced signaling is not restricted to the tumor site may be advantageous, as FTY720 treatment suggested that newly infiltrating cells from the periphery contribute to the antitumor activity of CD8-mIL2 (Supplementary Fig. S4C and S4D), consistent with other immunotherapies (68).

Next to the CD8-targeted IL2 described here, PD-1 has also been explored as an antigen for *cis*-targeting of IL2Rβγ agonists (42, 69). However, PD-1 expression has been documented in a variety of immune cells, including Tregs (70), myeloid cells (71, 72), γδ T cells (73), NK cells (74), and innate lymphoid cells (75). Treg activation is undesirable, and the roles of many of these other cell types in the context of IL2 therapy are unclear. Furthermore, as PD-1 expression is highest on terminally exhausted T cells (76), PD-1 targeting may bias activity toward PD-1^high^ exhausted cells, as compared with less dysfunctional PD-1^low/int^ T cells. In contrast, CD8β targeting by AB248 can provide broad targeting to all CD8^+^ T-cell subsets.

In summary, CD8^+^ T-cell restriction of IL2 signaling leads to strong antitumor activity in mice, robust pharmacodynamic effects in primates, and a favorable nonclinical safety profile. The data shown here demonstrate the potential of CD8^+^ T cell–selective IL2 to improve the therapeutic index of IL2, both as monotherapy and in combination with anti–PD-1. Collectively, these data demonstrate AB248’s differentiation from broadly acting IL2-based therapies and support AB248’s clinical development.

## Methods

### Generation of Proteins

For detailed descriptions of the molecules used in these studies, see Supplementary Table S1. The mouse not-α IL2 (CTRL-not-α-mIL2) was generated by fusing an antibody with no reactivity in mouse to a murine IL2 mutein lacking IL2Rα binding. The mouse CD8-mIL2 is a fusion between an anti-mouse CD8 mIgG2a and a single mouse IL2 mutein with no IL2Rα binding and reduced IL2Rβγ binding. The variable regions were generated via rat hybridoma and murinized via CDR grafting. For both mouse fusions, single IL2 loading was achieved via bispecific charge pair technology (77), and the IL2 mutein was fused to the C-terminus of one Fc chain via a flexible linker. To prevent FcγR-dependent effector function, mutations based on EU numbering L234A, L235A, and P329G were introduced into the CH2 domain of each heavy chain (78). AB248 is a fusion between an anti-human CD8αβ IgG1 and a single human IL2 mutein with no IL2Rα binding and reduced IL2Rβγ binding. The human not-α IL2 (CTRL-not-α-hIL2) is a fusion between an IgG1 and a single human IL2 mutein with no binding to IL2Rα. Single IL2 loading for all human IL2 fusion molecules was achieved via knob-in-hole (79), and mutations were introduced to prevent FcγR-mediated effector function. For all fusions, IL2 mutein was fused to the C-terminus of one Fc chain via a flexible linker. The mouse anti–PD-1 clone F12 is comprised of a murinized anti–PD-1 molecule with FcγR-null (D265A) mIgG1 Fc.

Proteins were expressed in HEK293 cells and then purified via affinity chromatography with protein A, followed by ion-exchange chromatography and size exclusion chromatography. Purity, integrity, and monomeric state of the fusion constructs were analyzed by SDS-PAGE and analytical size exclusion chromatography. The protein concentration of purified IL2 fusion constructs was determined by measuring the optical density at 280 nm.

Recombinant CD8αβ, comprising the extracellular domains of CD8α, fused to an acidic leucine zipper and an 8 × histidine tag, and CD8β, fused to a basic leucine zipper and a strep-tag II, was expressed in secreted form from HEK293 cells and sequentially purified by IMAC, size exclusion, and streptavidin-affinity column chromatography.

### Mouse Antitumor Activity Studies

The MC38, B16F10, and MCA205 mouse tumor efficacy studies were conducted using 6- to 10-week-old C57BL/6J female mice from Jackson Laboratories housed using the Innovive Disposable IVC Rodent Caging System in a 12-hour light/dark cycle. Protocols were approved by the Institutional Animal Care and Use Committees (IACUC) of Charles River Laboratories (Protocols EB17-010, EB17-010-116). Mice were injected s.c. into the right flank with either 1.5 × 10^6^ MC38c7 (kindly gifted from Washington University in St. Louis), 1.5 × 10^6^ MCA205 (ATCC SCC173), or 5.0 × 10^5^ B16F10 (ATCC CRL-6475). For d42m1-T3 sarcoma and KP.mLama4 tumor studies, 8- to 12-week-old male 129S6 mice or 129S4 mice were purchased from Taconic Farms and housed in a specific pathogen-free animal facility. These studies were performed in accordance with procedures approved by the AAALAC-accredited Animal Studies Committee of Washington University in St. Louis. Mice were injected s.c. into the flank with either 1.5 × 10^6^ d42m1-T3 or 1 × 10^6^ KP.mLama4 tumor cells (38). Mice were dosed intravenously once with treatment molecules as indicated. For the B7H3 CAR-T study, 5 × 10^6^ A375 tumors (ATCC) expressing the endogenous B7H3 antigen were subcutaneously implanted into the right flank of NOD.Cg-*Prkdc*^scid^*Il2rg*^tm1Wjl^/SzJ (NSG) mice from Jackson Laboratories. Following tumor engraftment for 11 days (average tumor size 65 mm^3^), 3.8 × 10^6^ anti-B7H3 CAR-T cells were intravenously (i.v.) injected into each mouse via the retro-orbital venous sinus followed by i.v. administration of either vehicle (PBS) or 0.5 mg/kg CD8-hIL2 molecule into the tail vein. Indicated subsequent doses of vehicle or CD8-hIL2 were delivered via the retro-orbital venous sinus. Tumors were assessed by standard caliper measurements and randomized one day prior to study initiation to yield groups with similar tumor volume. Tumor volume was calculated as follows: Volume = 0.5 × Width^2^ × Length. Tumor volumes and body weights were measured every 3 to 4 days throughout the study. Mice with tumor volumes exceeding 2,000 mm^3^ or body weight loss in excess of 20% of baseline were euthanized in accordance with approved IACUC protocols.

Studies requiring cellular depletion were conducted as follows: CD8^+^ T-cell depletion via anti-CD8β antibody (Bio X Cell clone 53-5.8) dosed 350 μg i.p. two days prior to therapy, on therapy day, and then weekly thereafter; CD4^+^ T-cell depletion via anti-CD4 antibody (Bio X Cell clone GK1.5) dosed 250 μg i.p. two days prior to therapy, on therapy day, and then weekly thereafter; and NK cell depletion via anti-NK1.1 (Bio X Cell clone PK136) 200 μg i.p. two days prior to therapy, on therapy day, and then weekly thereafter. Studies performed using FTY720 (Sigma-Aldrich SML0700) used 1 mg/kg FTY720 i.p. two days prior to therapy, on therapy day, and then twice weekly thereafter.

### Biodistribution in Mice

Biodistribution evaluations were performed in the peripheral blood, spleen, and tumor of C57BL/6J mice bearing B16F10 tumors. Treatment molecules were labeled utilizing the Alexa Fluor 647 Antibody Labeling Kit (Thermo Fisher) following the manufacturer’s instructions. The final degree of labeling for CD8-mIL2 and CTRL-not-α-mIL2 were 4.12 and 4.71 moles dye per mole protein, respectively. B16F10-bearing mice were dosed with 1 mg/kg of labeled CD8-mIL2 or CTRL-not-α-mIL2. After 24 hours, tissues were excised and, for spleen and tumor, manually dissociated by mincing and gently pushing pieces through a 70-μm cell strainer using a syringe plunger. After RBC lysis (eBioscience), cells were strained, counted, plated, and stained with a lineage panel, followed by fixation and analysis by flow cytometry.

### Generation of CAR-T Cells

Primary human T cells were isolated from buffy coats obtained from the Stanford Blood Center (Menlo Park) using the Human T-cell isolation kit (EasySep, cat no. 17951) following the manufacturer’s instructions. Isolated T cells (2 × 10^6^) were stimulated with human TransAct (Miltenyi) at 1:100 dilution in CTS OpTmizer media (Gibco) supplemented with 100 U/mL of recombinant human IL2 (PeproTech) in a 24-well non-tissue culture-treated plate (Corning) for 48 hours. Activated T cells (1 × 10^6^) were harvested and transduced with lentivirus that drove expression of the anti-B7H3 chimeric antigen receptor (CAR; ref. 80) in a 24-well G-Rex plate containing 3 mL of CTS OpTmizer media supplemented with IL2 (100 U/mL). CAR-T lentivirus was produced in HEK 293T cells. Transduced CAR-T cells were maintained in the G-Rex plate for 12 days by supplementation with additional media containing IL2 (100 U/mL) every two days. The B7H3 CAR-T cells were harvested on day 14 and frozen at 10 × 10^6^ CAR-T cells/mL in Bambanker freezing media (Bulldog Bio).

### Mouse *Ex Vivo* Immunophenotyping of Blood and TILs

Lymphocyte proliferation was assessed in peripheral blood and tumor samples from C57BL/6J mice bearing MC38 tumors treated from 12 to 14 days after tumor implantation. Excised tumors were chopped into ≤2 mm^3^ pieces, added to C-tubes (Miltenyi), and enzymatically dissociated using a mouse tumor dissociation kit (Miltenyi). Briefly, RPMI-1640 (Thermo) containing tumor dissociation enzymes as directed (except enzyme R, which was 1/20th listed concentration) was added to each tube. Tumors were then dissociated using gentleMACS Octo Dissociator (Miltenyi) program 37C_m_TDK_1 as directed by kit instructions. Once dissociation was complete, samples were placed on ice, washed with RPMI containing 5% FBS (Invitrogen), strained, and assessed for cell counts and viability. For each tumor sample, 1 × 10^7^ cells (or entirety of the sample if less) were transferred into a plate for staining. In a separate deep-well plate, 50 μL of EDTA-treated whole peripheral blood from each animal was added per well. Into each well of both plates, CountBright beads (Thermo) were added. In a subset of studies, tumor samples were resuspended in FACS buffer containing Rpl18 tetramer and incubated for 20 minutes on ice prior to surface staining. A staining mix containing FACS buffer, Brilliant Staining Buffer (BD Biosciences), Fixable Live/Dead (Thermo), and antibodies against surface markers was then added. After staining, samples were washed with FACS buffer and fixed. Tumor cells were fixed in PBS containing 4% paraformaldehyde (Thermo), whereas blood samples were resuspended in prewarmed BD Lyse/Fix buffer (BD Biosciences). After fixations, all samples were washed and resuspended in FACS buffer for analysis by flow cytometry. Biotinylated Rpl18 monomers were generously provided by the Schreiber lab and assembled via fluor-conjugated streptavidin in-house prior to use. T3 TIL staining was performed as follows: single-cell suspensions (1–4 million cells) in 50 μL of FACS buffer were incubated with APC-labeled mLama4 tetramer at 37°C for 20 minutes. Then, 50 μL of master mix containing surface antibodies, including fixable viability dye, was added to each sample and incubated for an additional 30 minutes at 4°C. Cells were washed twice with FACS buffer, and intracellular proteins were stained using a Foxp3/Transcription factor staining kit (Thermo Fisher) according to the manufacturer’s protocol. Rpl18- and mLama4-specific monomers were obtained from the Bursky Center Immunomonitoring Laboratory (IML) at Washington University School of Medicine in St. Louis and assembled via fluorconjugated streptavidin in-house prior to use.

### Binding Studies

Human PBMCs were plated at 300,000 cells per well in a 96-well plate, pelleted, and resuspended in 100 μL FACS buffer containing the indicated concentrations of antibody or fusion protein. The cells were incubated for 2 hours at 4°C, then washed with cold FACS buffer. Cells were then resuspended in FACS buffer containing surface-staining antibodies against the human Fc, then incubated for 30 minutes at 4°C protected from light. After staining, cells were washed with FACS buffer and then immediately fixed with 4% paraformaldehyde in PBS for 10 minutes at room temperature protected from light. Cells were washed once more and resuspended in FACS buffer for analysis by flow cytometry.

### ELISAs

The binding specificity of the antibodies was determined by ELISA. Recombinant CD8αβ, CD8α (Sino Biological), CD8β (Sino Biological), and ErbB2 (Sino Biological) at 1 μg/mL in PBS were coated onto a MaxiSorp plate (Invitrogen) overnight at 4°C. The plate was then blocked with a casein blocking solution (Thermo Fisher Scientific) for 2 hours at room temperature. After blocking, the plate was washed with wash buffer (PBS/0.05% Tween 20). Next, 97/47-IgG or OKT-8 (Invitrogen) at concentrations ranging from 30 to 0.0019 nmol/L in PBS/0.5% BSA/0.05% Tween 20 was added onto the plate and incubated for 1 hour at room temperature. After incubation, the plate was washed, and incubated with 1:3,000 dilution of anti-human IgG (Fc specific)–HRP conjugate or anti-mouse IgG (Fc specific)-HRP conjugate (Jackson ImmunoResearch) in PBS/0.5% BSA/0.05% Tween 20 for 1 hour. The plate was washed after incubation, and binding was detected by adding 1-Step Ultra TMB (Thermo Fisher Scientific) to the plate, followed by a stopping solution of 2 M sulfuric acid. Binding was measured in triplicate. Binding absorbance at 450 nm was measured using a Spectromax iD5 microplate spectrophotometer (Molecular Devices). For assessment of molecular stability following incubation with 10% human serum for 1 week at 37°C, the capture reagent was recombinant human CD8αβ, and 10 nmol/L of anti-human IL2 mouse IgG1 (R&D Systems), followed by anti-mouse IgG (Fc specific)-HRP to detect binding.

### Primary Cell Phospho-STAT5 and Ki67 Assays

STAT5 phosphorylation was assessed in human buffy coat from whole blood, heparinized cynomolgus whole blood (Worldwide Primates), and C57BL/6J mouse splenocytes. Briefly, blood or splenocytes were treated for 25 minutes with test molecules at 37°C, then immediately placed on ice and stained for surface markers. For whole blood assays, prewarmed BD Lyse/Fix (BD Biosciences) was added to each well and incubated at 37°C for 10 minutes. Alternatively, splenocytes were washed with chilled FACS buffer and then fixed with 4% paraformaldehyde in PBS. Following fixation, cells were washed and resuspended in chilled BD Perm Buffer III (BD Biosciences) and incubated for 1 hour at −20°C. Cells were then washed and stained for remaining surface and intracellular markers in eBioscience permeabilization buffer (Thermo). After washing, cells were resuspended in FACS buffer and analyzed by flow cytometry.

To assess Ki-67 upregulation in human PBMCs, cells were plated at 200,000 per well and incubated in complete media [RPMI-1640 (Gibco) containing 10% FBS (Invitrogen), Glutamax (2 mmol/L, Gibco), Na Pyruvate (1 mmol/L, Gibco), MEM NEAA (1×, Gibco), HEPES (10 mmol/L, Gibco), and 2-mercaptoethanol (50 μmol/L, Gibco)] containing test molecules for 120 hours. After incubation, cells were stained for viability and surface markers followed by fixation and permeabilization using eBioscience FoxP3/Transcription Factor Staining Buffer Set (Thermo). Cells were then stained for intracellular markers and analyzed by flow cytometry.

### RNA-Sequencing Analysis of Stimulated Human CD8^+^ T Cells

Human PBMCs from 3 donors were sorted into the following subsets based on the expression of CD45RO and CD62 L markers: naive CD8^+^ T cells (CD62L^+^CD45RO^−^), central memory CD8^+^ T cells (CD62L^+^CD45RO^+^), effector memory CD8^+^ T cells (CD62L^−^CD45RO^+^), and effector CD8^+^ cells (CD62L^−^CD45RO^−^). Briefly, 70,000 to 200,000 cells were added to 96-well plates in 100 μL AIM V medium (Thermo). Cells were stained with antibodies, sorted, and collected in AIM V medium (Gibco) and then treated at concentrations corresponding to the EC95 by pSTAT5 of rhIL2 (R&D Systems) or AB248 for 24 hours. At the end of stimulation, cells were pelleted, and RNA was isolated per the manufacturer’s instructions (Arcturus PicoPure RNA Isolation Kit, Applied Biosystems). RNA was sent to Genewiz for sequencing using the Ultra-Low Input RNA-seq protocol. Sequencing data were aligned against the human reference genome (Ensembl GrCh38) using the STAR software package and quantified using the featureCounts package. Differential expression analysis was performed using the DESeq2 Bioconductor package.

### T-cell Coreceptor Antigen Recognition Assay Using CMV-Reactive T Cells

T2 cells (ATCC, cat no. CRL-1992) were cultured untreated or pulsed overnight with 50 nmol/L of HLA-A2 restricted CMV pp65 peptide (Cellero) at 37°C in 5% CO_2_. The following day, cells were labeled with CellTrace Violet (Thermo) and plated at 10,000 cells per well in complete media. CMV-reactive T cells (Donor 401, Cellero) were thawed, counted, and plated at 30,000 cells per well. Finally, treatment molecules were added, and cells were incubated for 24 hours at 37°C in 5% CO_2_. After 24 hours, supernatants were harvested for analysis by IFNγ ELISA (Thermo) per the manufacturer’s instructions.

### Human Primary Cell Cytokine Release Assays

PBMCs were isolated from the blood of healthy human donors using Ficoll-Paque Plus (GE Healthcare) and red blood cells were lysed using ACK lysis buffer (Gibco) according to the manufacturer’s instructions. For the NK depletion, a subset of the PBMCs were incubated with anti-CD56 microbeads (Miltenyi), then separated through a magnetic column per the manufacturer’s directions. For both whole PBMCs and NK-depleted PBMCs, 500,000 cells per well were plated in complete media. Treatment molecules were then added to each well and the plate was incubated for 24 hours at 37°C with 5% CO_2_. Supernatants were harvested for analysis by meso scale discovery (MSD).

For isolated cell subset studies, NK cells and pan T cells were isolated by negative selection using magnetic beads (StemCell) and were subsequently stained for surface markers for 20 minutes at 4°C prior to flow cytometry sorting. Sorted cell subsets included NK cells (CD3^−^CD14^−^CD19^−^CD56^+^), CD4^+^ T cells (CD14^−^CD19^−^CD56^−^CD3^+^CD4^+^CD8^−^), and CD8^+^ T cells (CD14^−^CD19^−^CD56^−^CD3^+^CD4^−^CD8^+^). All cells were plated at 50,000 cells per well in complete media with treatment molecules. After a 24-hour incubation, supernatants were harvested for analysis by MSD. Results were normalized on a per-cell basis for visualization when necessary.

### Assessment of Human CD8^+^ T Cells in the Presence of CD3/CD28 Stimulation

CD8^+^ T cells were isolated from healthy human donors by negative selection using magnetic beads (StemCell) and were subsequently plated at 200,000 cells per well in complete media containing Trans-Act (1:10,000 final dilution, Miltenyi) and treatment molecules. Cells were cultured for 2 days at 37°C with 5% CO_2_, at which point supernatants were harvested for analysis by 5-plex MSD.

### Flow Cytometry

Human flow cytometry included antibodies against the following: CD3, CD4, CD8α, CD8β, CD25, CD127, CD56, CD16, CD19, hFc, CD14, Perforin, FoxP3, pSTAT5, CD45RO, CD62L, Ki-67, gamma delta TCR, CD122 (IL-2Rb), and CD132 (IL-2Rg). In IL2R quantification studies, BD Quantibrite Beads (BD Biosciences) were used. Receptor number calculations were performed following the manufacturer’s instructions.

Cynomolgus monkey flow cytometry included antibodies targeting the following: CD3, CD4, CD8α, CD8β, CD25, CD16, CD159a (NKG2A), gamma delta TCR, CD14, hFc, HLA-DR, granzyme B, Perforin, Ki-67, and FoxP3.

Mouse flow cytometry antibodies included: CD3, CD4, CD8α, CD8β, CD25, NK1.1, NKp46, CD49b, CD19, hFc, CD14, FoxP3, pSTAT5, CD44, CD62L, Ki-67, gamma delta TCR, 41BB, PD-1, TIM3, CD90.2, GZMB, LAG3, TOX as well as Rpl18 and mLama4 tetramers. Absolute cell counts were assessed using CountBright Absolute Counting Beads (Thermo Fisher).

Additional details about the antibodies used can be found in Supplementary Table S2.

All samples were acquired in FACS buffer (containing PBS with 2 mmol/L EDTA and 0.5% BSA or 2% FBS) on a CytoFLEX LX (Beckman Coulter) or LSRFortessa X-20 (BD Biosciences, cynomolgus monkey study only), and analyzed with FlowJo v10.8.0 software.

### Single-cell RNA, Protein, and TCR Sequencing Analyses of TILs

For the scRNA-seq performed in T3 sarcoma, T3 tumor-bearing mice were treated on day 12 after tumor transplant. Two and 4 days later, single-cell suspensions were prepared from TILs. Total CD8^+^ T cells were enriched using a CD8^+^ T cell–positive selection kit (Miltenyi). Tumors from individual mice were labeled with one of the following antibodies before pooling in order to identify cells from individual tumors in downstream analysis: (i) TCRβ (TotalSeq-C0120 anti-mouse TCR β chain antibody, cat no. 109259, BioLegend), (ii) CD90.2 (TotalSeq-C0075 anti-mouse CD90.2 (Thy1.2) antibody, cat no., 105353, BioLegend), and (iii) CD45 (TotalSeq-C0096 anti-mouse CD45 antibody, cat no. 103169, BioLegend). Samples were submitted to the Genome Institute at Washington University in St. Louis to generate 10× libraries using 10 × 5′v2 Single-Cell RNA-seq, V(D)J enrichment, feature barcoding, and Barcode Enabled Antigen Mapping kits.

Fastq files were processed using the Cell Ranger (v7.1.0) multi pipeline with the default parameters. Gene-expression libraries were aligned to the mouse transcriptome (mm10-2020-A) and TCR sequences were aligned to the VDJ reference data set (GRCm38-alts-ensembl-5.0.0). Feature barcode and BEAM conjugates used in this study are listed in Supplementary Table S3.

Cells with less than 7500 detected unique molecular identifiers (UMI) or greater than 5% mitochondrial genes were removed from the analysis. To identify CD8^+^ subpopulations, principal component analysis was performed on highly variable genes (FDR < 0.05), and the first 50 principal components were used for nearest neighbor calculations, Louvain clustering (k = 25), and Uniform Manifold Approximation and Projection (UMAP) visualization. To remove the effects of proliferation on cell-type identification, genes correlated (*P* > 0.2) with either *Mki67*, *Top2a*, *Hist1h2ap*, or *Hist1h1e* were excluded from the set of highly variable genes before running all dimensionality reduction and clustering algorithms. After clustering, one cluster with low *Cd8b1* and *Cd8a* expression was removed and cells were reclustered for subsequent analyses.

To calculate gene-set enrichment scores, cluster marker genes were first identified using a Wilcoxon test implemented in the findMarkers function from the scran R package (v1.26.2). Gene-set enrichment analysis (GSEA) of cluster markers clusters was calculated using the fgsea R package (v1.24.0). The proliferating, effector-like, and progenitor-exhausted signatures were taken from Miller and colleagues (44). The early-effector-like, intermediate exhausted, and intermediate/terminal exhausted signatures were taken from Pauken and colleagues (41). The naive and memory CD8^+^ T-cell signatures were taken from the molecular signature database (MSigDB; ref. 43). The stem-like and migration signatures were taken from Deak and colleagues (42), and the better effector signature was derived from a reanalysis of scRNA-seq data, available from ArrayExpress with accession number E-MTAB-11773.

scRNA-seq differential expression between treatment conditions was performed by pseudobulking counts from individually hash-tagged mice. Differential expression analysis was then performed using the DESeq2 Bioconductor package.

Single-cell antigen specificities were assigned in a two-step approach. In the first step, the median antigen specificity score was calculated for each CD8 clonotype and each BEAM conjugate. For clonotypes with greater than 3 cells, all cells within the clonotypes were assigned antigen specificities if the median specificity score was greater than 30. In cases where a clonotype was assigned multiple antigen specificities, the antigen with a higher median specificity score was used. In the second step, all cells were labeled as antigen-specific if the specificity score was greater than 90. For individual cells assigned multiple antigen specificities, a single specificity was assigned using the highest specificity score, highest number of antigen UMIs, and highest number of antigen reads. In cases where the specificity assigned based on clonotype differed from the single-cell assignment, the clonotype specificity was used.

For the MC38 study, mice bearing MC38 tumors were randomized and dosed with treatment molecules as indicated 14 days after tumor implantation. Three days after treatment, single-cell suspensions were made from excised tumors using enzymatic dissociation as previously described. Individual tumors were then stained with sorting (Calcein AM, PI, and CD45) as well as Totalseq (CD45.1, CD45.2, TCRβ, TCR γ/δ, CD8a, CD4, and PD-1) antibodies. Sorted PI^−^/CalceinAM^+^/ CD45^+^ cells from each tumor were pooled by treatment group. To enable individual mouse deconvolution, pooled samples for library preparation consisted of cells labeled by either CD45.1 or CD45.2. Samples were submitted to SeqMatic to generate 10x libraries using 10 × 5′v2 Single-Cell RNA-seq, V(D)J enrichment and feature barcoding.

Analysis was performed as described above for CD8^+^ TILs with the following differences: Cells with fewer than 300 detected genes or greater than 5% mitochondrial genes were removed from the analysis. After an initial clustering of total CD45^+^ cells, CD8^+^ and myeloid clusters were manually identified and reclustered using parameter values of k = 5 and k = 25 for Louvain clustering, respectively.

### Nonhuman Primate Studies

Cynomolgus monkey studies were conducted by Shin Nippon Biomedical Laboratories, Ltd. (SNBL) in accordance with IACUC guidelines (Approval Nos. IACUC551-001, IACUC551-003, and IACUC551-005) and was performed in accordance with the animal welfare bylaws of SNBL Drug Safety Research Laboratories, which is accredited by AAALAC International. The studies were conducted in accordance with the Animal Welfare Act, the Guide for the Care and Use of Laboratory Animals, and the Office of Laboratory Animal Welfare. Cynomolgus monkeys were dosed intravenously with AB248 and peripheral blood was isolated and analyzed for immune cell counts via hematology and flow cytometry. Absolute counts were determined by relating the percentage of each lymphocyte population as determined by flow cytometry to the absolute lymphocyte counts as determined by hematology using the Fully Automated Hematology Analyzer (Sysmex Corporation).

### Statistical Analysis and Reproducibility

The number of replicates in each experiment and the number of independent experimental runs are indicated in the figure legends. Statistical methods were not used to determine sample sizes for animal studies; sample sizes for animal experiments were estimated based on variability observed in pilot studies. In murine studies, treatment and control animals were randomized according to tumor volume immediately prior to the start of treatment, and studies were not performed in a blinded fashion. No animals were excluded from the analysis.

Statistical analyses were performed using GraphPad Prism software v.10.0.0 for macOS. Where multiple comparisons were performed, preplanned analysis used one-way ANOVA with Dunnett multiple comparisons test. *P* < 0.05 was used as a cutoff for statistical significance. Curve fits for *in vitro* pSTAT5, Ki-67, and binding studies were performed using GraphPad Prism software v.10.0.0 for macOS using nonlinear fit of [Agonist] vs. response (3 parameters).

### Data Availability

Source data are provided in this paper.

Raw and processed bulk RNA-sequencing data from the human CD8^+^ T cells have been deposited in the Gene-Expression Omnibus (GEO) under accession number GSE24360. Raw and processed sequencing data from the scRNA-seq mouse tumor study have been deposited in GEO under accession number GSE252650. Other data supporting this report are available from the corresponding author upon reasonable request.

## Supplementary Material

Supplementary Table S1 Supplementary Table S1, Molecules used in this study

Supplementary Table S2 Supplementary Table S2, Antibodies used in this study

Supplementary Table S3 Supplementary Table S3, Single-cell RNA sequencing reagents used in this study

Supplementary Figure S1 Supplementary Figure S1: CD8+ T cells drive anti-tumor activity but NK cells are responsible for toxicity with not-α-IL2 therapy.

Supplementary Figure S2 Supplementary Figure S2: In vitro culture of human CD8+ T cells, evaluation of CD8α and CD8β expression, and cytokine release assessment.

Supplementary Figure S3 Supplementary Figure S3: Biodistribution of cytokine fusion molecules in tumor-bearing mice.

Supplementary Figure S4 Supplementary Figure S4: Further characterization of CD8-mIL2 activity in mouse tumor models.

Supplementary Figure S5 Supplementary Figure S5: Anti-tumor activity of CD8-mIL2 in combination with anti-PD-1 in B16F10, 1956, MCA-205, and KP.mLama4 tumor models.

Supplementary Figure S6 Supplementary Figure S6: Characterization of antigen-specific CD8+ T cells in MC38 tumors.

Supplementary Figure S7 Supplementary Figure S7: Sub-clustering of naïve-like/recently activated cluster and characterization of clonal expansion and antigen.

Supplementary Figure S8 Supplementary Figure S8: Differential expression by treatment.

Supplementary Figure S9 Supplementary Figure S9: scRNAseq analysis of MC38 tumors.

Supplementary Figure S10 Supplementary Figure S10: In vitro and in vivo activity of AB248 in cynomolgus monkey.
