# Supplementary material for: IL2 Targeted to CD8+ T Cells Promotes Robust Effector T-cell Responses and Potent Antitumor Immunity
Source: Cancer Discov. 2024 Apr 9;14(7):1206–25. doi: 10.1158/2159-8290.CD-23-1266 (PMC11215410; doi:10.1158/2159-8290.CD-23-1266)
Supplement: Supplementary Table S1 — , Molecules used in this study [file cd-23-1266_supplementary_table_s1_suppst1.pdf]

Supplementary Table S1, Molecules used in this study

**Molecule**	**Description**	**Fc**	**IL-2**	**IL-2Rα binding**	**IL-2Rβγ binding**
AB248 (or CD8-IL2β*γ)	Anti-human CD8β antibody (clone 97/47) fused to single mutein via flexible glycine-serine linker.	hIgG1 with knob-in-hole mutations[83] used for single IL-2 fusion; L234A, L235A, and G237A mutations to abolish FcγR interactions	human	none	reduced
CD8-wtIL2			human	wild type	wild type
CD8-IL2βγ			human	none	wild type
CD8-IL2αγ			human	wild type	reduced
CD8α-IL2β*γ	Anti-human CD8α antibody (humanized clone OKT8) fused to single mutein via flexible glycine-serine linker.		human	none	reduced
CD8-hIL2	Anti-human CD8β antibody (clone 177/2) fused to single mutein via flexible glycine-serine linker.		human	none	reduced
CTRL-wtIL2	Fc fusion to single IL-2 mutein via flexible glycine-serine linker.		human	wild type	wild type
CTRL-IL2βγ			human	none	wild type
CTRL-IL2αγ			human	wild type	reduced
CTRL-IL2β*γ			human	none	reduced
CTRL-not-α-hIL2	Anti-human FAP antibody fused to single mutein via flexible glycine-serine linker.		human	none	wild type
CD8-mIL2	Anti-mouse CD8β antibody fused to single mutein via flexible glycine-serine linker.	mIgG2a with bispecific charge mutations[81] and L234A, L235A, P329G mutations to abolish FcgR interactions	mouse	none	reduced
CTRL-not-α-mIL2	Anti-human CD8β antibody (no affinity for mouse CD8β) fused to single mutein via flexible glycine-serine linker.		mouse	none	wild type
