# Supplementary material for: IL2 Targeted to CD8+ T Cells Promotes Robust Effector T-cell Responses and Potent Antitumor Immunity
Source: Cancer Discov. 2024 Apr 9;14(7):1206–25. doi: 10.1158/2159-8290.CD-23-1266 (PMC11215410; doi:10.1158/2159-8290.CD-23-1266)
Supplement: Supplementary Table S2 — , Antibodies used in this study [file cd-23-1266_supplementary_table_s2_suppst2.pdf]

Supplementary Table S2, Antibodies used in this study

Species	Target	Fluor	Clone	Vendor	Catalog #
Human	CD3	PerCPCy5.5	UCHT1	BioLegend	300430
Human	CD4	PacBlue	RPA-T4	BioLegend	300521
Human	CD4	FITC	RPA-T4	BioLegend	300538
Human	CD8a	BV510	SK1	BioLegend	301048
Human	CD16	BV650	3G8	BioLegend	302042
Human	CD45RO	BV510	UCHL1	BioLegend	304246
Human	CD62L	A700	DREG-56	BioLegend	304820
Human	CD62L	BV421	DREG-56	BioLegend	304828
Human	41BB	PE	4B4-1	BioLegend	309804
Human	CD56	PE	HCD56	BioLegend	318306
Human	PD-1	PE	EH12.2H7	BioLegend	329906
Human	PD-1	BV786	EH12.2H7	BioLegend	329930
Human	TCRgd	PEDazzle594	B1	BioLegend	331226
Human	CD45RO	PECy7	UCHL1	BioLegend	337168
Human	IL2RG	PE	TUGH4	BioLegend	338606
Human	CD161	BV786	HP-3G10	BioLegend	339930
Human	CD56	PE	NCAM16.2	BD Biosciences	340363
Human	CD127	BV605	A019D5	BioLegend	351334
Human	Perforin	APCFire750	B-D48	BioLegend	353318
Human	CD25	APC	M-A251	BioLegend	356110
Human	CD25	BV421	M-A251	BioLegend	356114
Human	CD56	KIRAVIA Blue 520	5.1H11	BioLegend	362566
Human	CD4	FITC	L200	BD Biosciences	550628
Human	IL2RB	PE	MIK-B3	BD Biosciences	554525
Human	CD3	APC	UCHT1	BD Biosciences	555335
Human	CD14	APC	M5E2	BD Biosciences	555399
Human	CD19	APC	HIB19	BD Biosciences	555415
Human	CD69	FITC	FN50	BD Biosciences	555530
Human	CD3	APC/Cy7	SP34-2	BD Biosciences	557757
Human	Ki67	BUV395	B56	BD Biosciences	558615
Human	FoxP3	PECF594	259D	BD Biosciences	562421
Human	TCRgd	PECF594	B1	BD Biosciences	562511
Human	CD3	BUV395	UCHT1	BD Biosciences	563546
Human	41BB	BV421	4B4-1	BD Biosciences	564091
Human	CD4	BB515	RPA-T4	BD Biosciences	564419
Human	CD45RO	BB515	UCHL1	BD Biosciences	564529

Human	CD25	BB700	M-A251	BD Biosciences	566447
Human	CD56	R718	HCD56	BD Biosciences	566965
Human	CD4	BUV805	RPA-T4	BD Biosciences	569196
Human	CD14	BUV737	M5E2	BD Biosciences	612763
Human	CD56	BUV737	NCAM16.2	BD Biosciences	612767
Human	CD8a	BUV805	SK1	BD Biosciences	612889
Human	CD4	BV786	L200	BD Biosciences	740996
Human	CD56	R718	NCAM16.2	BD Biosciences	751984
Human	pSTAT5	PE	A17016B.Rec	BioLegend	936904
Human	pSTAT5	APC	A17016B.Rec	BioLegend	936906
Human	FoxP3	PECy7	236A/E7	eBiosciences	25-4777-42
Human	CD8b	PerCP-eFluor 710	SIDI8BEE	eBiosciences	46-5273-42
Human	CD8b	e660	SIDI8BEE	eBiosciences	50-5273-42
Human	TCR γ/δ	BV421	B1	BioLegend	331218
Cynomolgus monkey	CD14	PE-Cy7	M5E2	BD Biosciences	557742
Cynomolgus monkey	CD14	BV650	M5E2	BD Biosciences	563420
Cynomolgus monkey	CD16	BV650	3G8	BioLegend	302042
Cynomolgus monkey	CD25	BB700	M-A251	BD Biosciences	566447
Cynomolgus monkey	CD3	APCCy7	SP34-2	BD Biosciences	557757
Cynomolgus monkey	CD4	BV786	L200	BD Biosciences	563914
Cynomolgus monkey	CD8a	BV421	SK1	BioLegend	344732
Cynomolgus monkey	CD8b	e660 (APC)	SIDI8BEE	eBiosciences	50-5273-42
Cynomolgus monkey	FoxP3	PE-eFluor610	PCH101	Thermo	61-4776-42
Cynomolgus monkey	gdTCR	PE	B1	BD Biosciences	561994
Cynomolgus monkey	Granzyme	AlexaFluor700	GB11	BD Biosciences	560213
Cynomolgus monkey	HLA-DR	AlexaFluor700	L243	BioLegend	307626
Cynomolgus monkey	Ki67	AlexaFluor647	B56	BD Biosciences	558615
Cynomolgus monkey	NKG2A	FITC	REA110	Miltenyi	130-113-565
Cynomolgus monkey	NKG2A	Pe-Vio770	REA110	Miltenyi	130-113-567
Cynomolgus monkey	Perforin	FITC	PF-344	Mabtech	3465-7
Mouse	41BB	PE	4B4-1	BioLegend	309804
Mouse	CD19	BUV737	1D5	BD Biosciences	612781
Mouse	CD19	A700	6D5	BioLegend	115528
Mouse	CD19	BV421	6D5	BioLegend	115538
Mouse	CD25	PE	7D4	BD Biosciences	558642
Mouse	CD25	PECy7	PC61	BioLegend	102016
Mouse	CD25	PerCPCy5.5	PC61	BioLegend	102030
Mouse	CD3	FITC	145-2C11	BioLegend	100306
Mouse	CD3	PerCPCy5.5	17A2	BioLegend	100218
Mouse	CD4	BUV359	GK1.5	BD Biosciences	563790

Mouse	CD4	Pacific Blue	GK1.5	BioLegend	100428
Mouse	CD44	BV510	IM7	BioLegend	103044
Mouse	CD45	BUV805	30-F11	BD Biosciences	568336
Mouse	CD49b	FITC	DX5	BD Biosciences	553857
Mouse	CD62L	APC	MEL-14	BioLegend	104412
Mouse	CD8a	BV510	53-6.7	BioLegend	100714
Mouse	CD8a	APCFire750	53-6.7	BioLegend	100766
Mouse	GZMB	PECY5	QA16A02	BioLegend	372226
Mouse	LAG3	BV650	C9B7W	BioLegend	125227
Mouse	NK1.1	BV605	PK136	BioLegend	108740
Mouse	PD-1	BV786	RMP1-30	BD Biosciences	748264
Mouse	pSTAT5	AlexaFluor647	47/Stat5(pY694)	BD Biosciences	562076
Mouse	TIM3	BV421	B8.2C12	BioLegend	134019
Mouse	TOX	PE	12-6502-82	eBiosciences	12-6502-82
