# Supplementary material for: IL2 Targeted to CD8+ T Cells Promotes Robust Effector T-cell Responses and Potent Antitumor Immunity
Source: Cancer Discov. 2024 Apr 9;14(7):1206–25. doi: 10.1158/2159-8290.CD-23-1266 (PMC11215410; doi:10.1158/2159-8290.CD-23-1266)
Supplement: Supplementary Table S3 — , Single-cell RNA sequencing reagents used in this study [file cd-23-1266_supplementary_table_s3_suppst3.pdf]

Supplementary Table S3, Single-cell RNA sequencing reagents used in this study

Category	Target	Oligonucleotide tag	Clone	Vendor	Catalog #
Surface protein	CD45	TGGCTATGGAGCAGA	30-F11	BioLegend	103169
Surface protein	CD90.2	CCGATCAGCCGTTTA	30-H12	BioLegend	105353
Surface protein	TCRb	TCCTATGGGACTCAG	H57-597	BioLegend	109259
BEAM	control	CCGTCTCACCGATAT	-	10x Genomics	1000546
BEAM	control	CGGCTCACCGCGTCT	-	10x Genomics	1000546
BEAM	mLama4	CTATCTACCGGCTCG	-	10x Genomics	1000546
BEAM	mLama4	CATGTCTACGTTAAG	-	10x Genomics	1000546
BEAM	mLama4	GAGGTACACCAATCA	-	10x Genomics	1000546
BEAM	mLama4	AGCACGACCTTGGTT	-	10x Genomics	1000546
BEAM	mLama4	TATCCATATACAGGA	-	10x Genomics	1000546
BEAM	mLama4	AATAATCTTGCGCTT	-	10x Genomics	1000546
BEAM	mLama4	CTTGCATGTAATGTA	-	10x Genomics	1000546
BEAM	mLama4	CGTCCTCGACTGTCC	-	10x Genomics	1000546
