# Supplementary material for: IL2 Targeted to CD8+ T Cells Promotes Robust Effector T-cell Responses and Potent Antitumor Immunity
Source: Cancer Discov. 2024 Apr 9;14(7):1206–25. doi: 10.1158/2159-8290.CD-23-1266 (PMC11215410; doi:10.1158/2159-8290.CD-23-1266)
Supplement: Supplementary Figure S1 — CD8+ T cells drive anti-tumor activity but NK cells are responsible for toxicity with not-α-IL2 therapy. [file cd-23-1266_supplementary_figure_s1_suppsf1.pdf]

Supplementary Figure S1

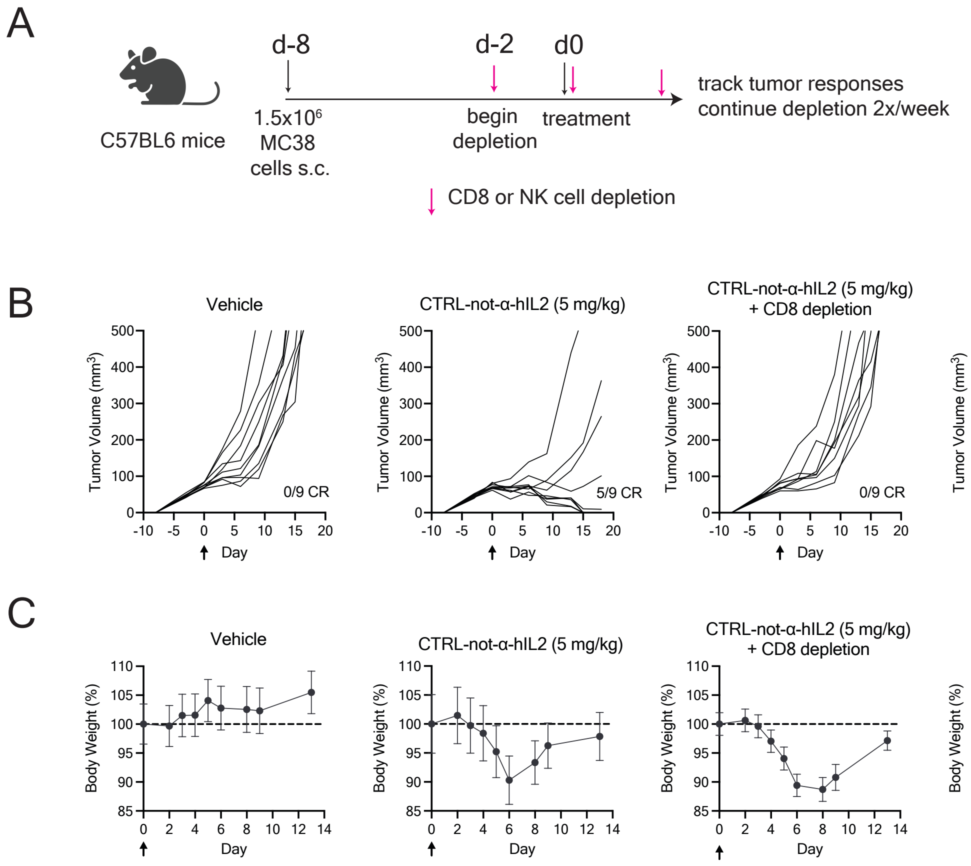

Supplementary Figure S1: CD8⁺ T cells drive anti-tumor activity but NK cells are responsible for toxicity with not-α-IL2 therapy. A-C, C57BL6 mice implanted with MC38 s.c. tumors were treated once with not-α human IL-2 (CTRL-not-α-hIL2) at the indicated doses. Mice were depleted of $CD8^+$ T cells or NK cells beginning 2 days prior to therapy and throughout therapy **A**, Schematic of the experimental design. Tumor size (**B**) and body weight (**C**) were assessed after treatment (n=9 per group).
