# Supplementary material for: IL2 Targeted to CD8+ T Cells Promotes Robust Effector T-cell Responses and Potent Antitumor Immunity
Source: Cancer Discov. 2024 Apr 9;14(7):1206–25. doi: 10.1158/2159-8290.CD-23-1266 (PMC11215410; doi:10.1158/2159-8290.CD-23-1266)
Supplement: Supplementary Figure S2 — In vitro culture of human CD8+ T cells, evaluation of CD8α and CD8β expression, and cytokine release assessment. [file cd-23-1266_supplementary_figure_s2_suppsf2.pdf]

Supplementary Figure S2

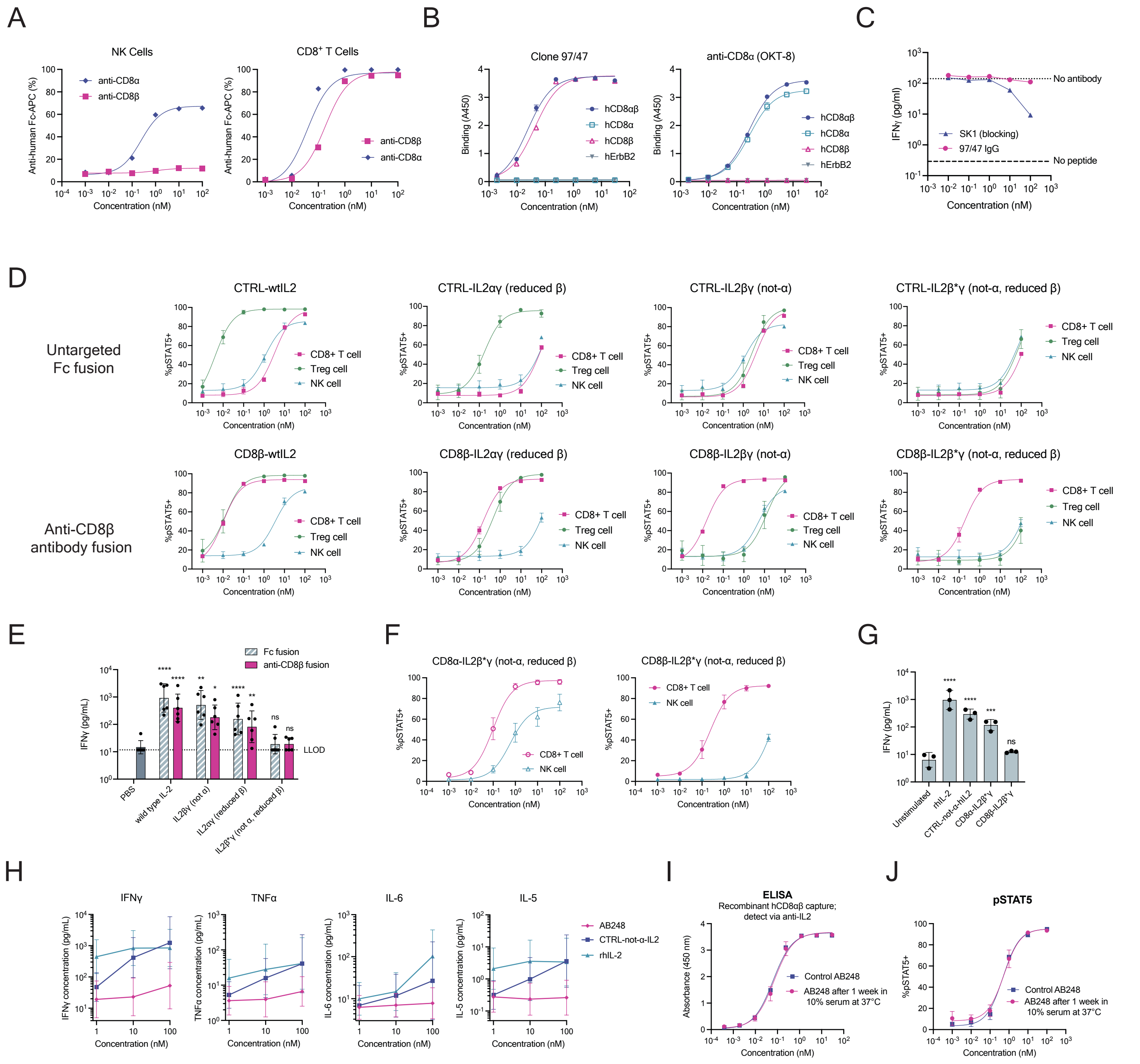

Supplementary Figure S2: In vitro culture of human CD8$^+$ T cells, evaluation of CD8α and CD8β expression, and cytokine release assessment. **A**, Binding of CD8$^+$ T cells or NK cells from human PBMCs using a CD8β-targeting antibody (clone 97/47) or CD8α (clone SK1, BD Biosciences). **B**, ELISA to evaluate binding to recombinant human CD8α, CD8β, or CD8αβ. Shown is binding for clone 97/47 (left) or the anti-CD8α antibody OKT8 (right). Recombinant human ErbB2 was used as a negative control. **C**, CMV pp65-reactive CD8$^+$ T cells were cultured in the presence of pp65 peptide-pulsed T2 cells with the indicated concentrations of antibody clone 97/47 or control blocking clone SK1 for 24 h, and IFN-γ was measured in supernatant by ELISA. **D**, pSTAT5 was assessed by flow cytometry following a 25-minute stimulation of human blood with the indicated untargeted Fc fusions (top row) or CD8β-targeted fusions (bottom row) (n=3). **E**, PBMCs were cultured for 24 hours in the presence of the indicated fusions and IFN-γ was quantified in the supernatant using MSD (n=6). **F**, pSTAT5 was assessed by flow cytometry following a 25-minute stimulation of human blood with the indicated molecules (n=2). **G**, PBMCs were cultured for 24 hours in the presence of the indicated fusions and IFN-γ was quantified in the supernatant using MSD (n=3). **H**, Human PBMCs were cultured in the presence of the indicated concentrations of rhIL-2, CTRL-not-α-hIL2, or AB248 for 24 h, and supernatant levels of IFN-γ, TNF-α, IL-6, and IL-5 were assessed by MSD (n=15 donors). **I-J**, AB248 was incubated in the presence of 10% human serum at 37°C for 1 week and assayed for molecule intactness. **I**, ELISA with recombinant human CD8αβ capture and anti-IL2 detection. **J**, pSTAT5 was assessed by flow cytometry following a 25-minute stimulation of human blood. Plotted are geometric mean values ± geometric s.d. for **E** and **G** and mean ± s.d. for **D**, **F, I, and J**. Studies are representative of 2-3 independent experiments. Statistics performed via one-way ANOVA with Dunnett's multiple comparisons test versus control (n.s., $P > 0.05$, $^*P < 0.05$, $^{**}P < 0.01$, $^{***}P < 0.001$, $^{****}P < 0.0001$).
