# Supplementary material for: IL2 Targeted to CD8+ T Cells Promotes Robust Effector T-cell Responses and Potent Antitumor Immunity
Source: Cancer Discov. 2024 Apr 9;14(7):1206–25. doi: 10.1158/2159-8290.CD-23-1266 (PMC11215410; doi:10.1158/2159-8290.CD-23-1266)
Supplement: Supplementary Figure S3 — Biodistribution of cytokine fusion molecules in tumor-bearing mice. [file cd-23-1266_supplementary_figure_s3_suppsf3.pdf]

Supplementary Figure S3

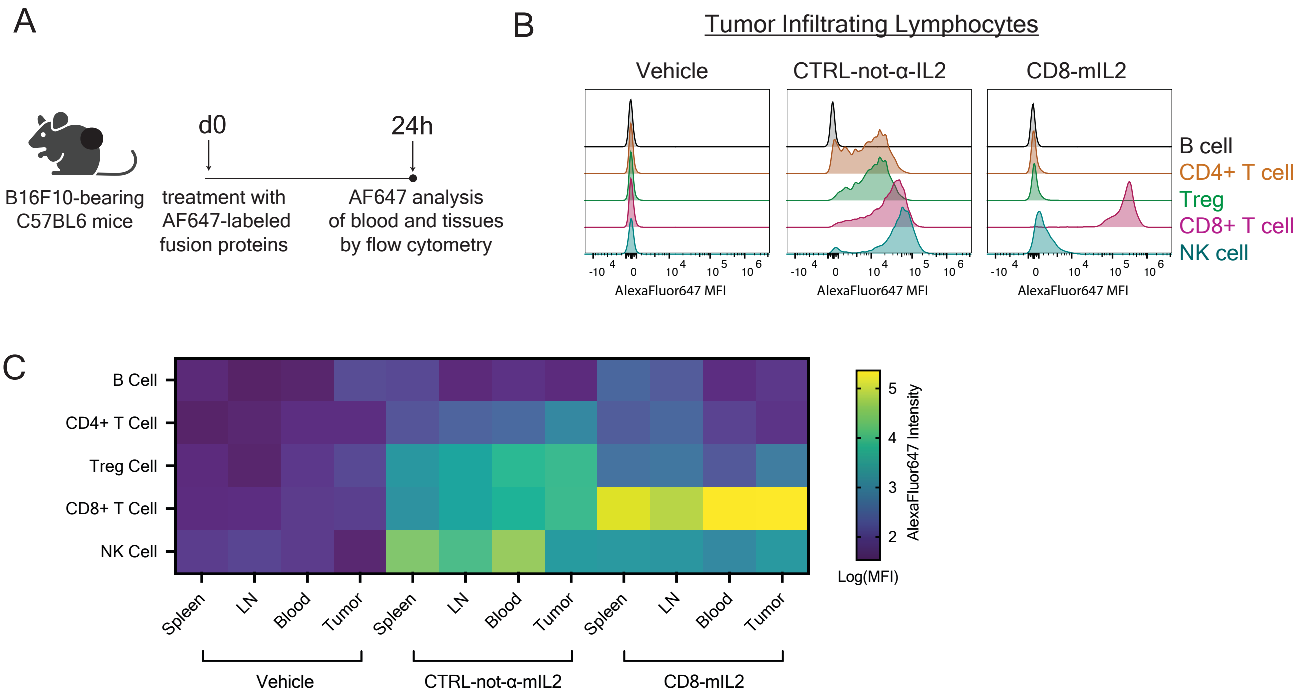

Supplementary Figure S3: Biodistribution of cytokine fusion molecules in tumor-bearing mice. A, B16F10-bearing C57BL6J mice were injected intravenously with 1 mg/kg of AlexaFluor647-labeled CD8-mIL2 or CTRL-not-α-mIL2 and 24 h later spleen, inguinal lymph nodes, blood, and tumor were isolated and analyzed by flow cytometry. Shown are representative flow plots assessing AlexaFluor647 intensity in TIL populations in a representative animal (**B**), and (**C**), a heatmap quantitating average AlexaFluor647 MFI across all cell types and tissues analyzed (n=2 mice per condition). Study is representative of two independent experiments.
