# Supplementary material for: IL2 Targeted to CD8+ T Cells Promotes Robust Effector T-cell Responses and Potent Antitumor Immunity
Source: Cancer Discov. 2024 Apr 9;14(7):1206–25. doi: 10.1158/2159-8290.CD-23-1266 (PMC11215410; doi:10.1158/2159-8290.CD-23-1266)
Supplement: Supplementary Figure S4 — Further characterization of CD8-mIL2 activity in mouse tumor models. [file cd-23-1266_supplementary_figure_s4_suppsf4.pdf]

Supplementary Figure S4

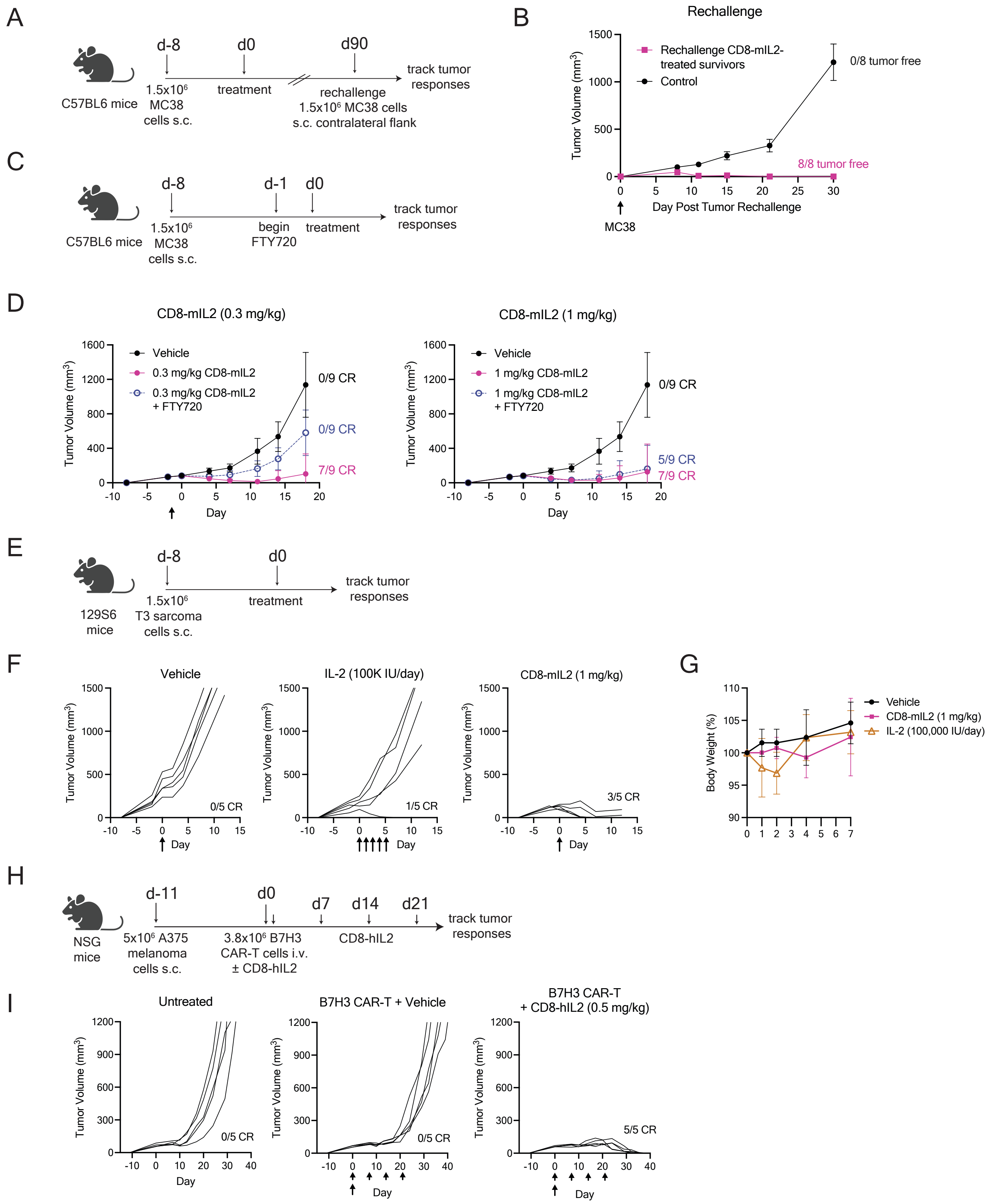

Supplementary Figure S4: Further characterization of CD8-mIL2 activity in mouse tumor models. **A**, C57BL6 mice that successfully rejected MC38 tumors with CD8-mIL2 therapy or age-matched control mice were rechallenged 90 days after primary tumor implant. Shown is a schematic of the experimental design (**A**), and tumor size (**B**) (n=8). **C-D,** C57BL6 mice implanted with MC38 s.c. tumors were treated once with CD8-mIL2 with or without concurrent FTY720 treatment (n=9). **E-G**, 129S6 mice were implanted with d42m1-T3 sarcoma cells and treated 8 days later with a single dose of 1 mg/kg of CD8-mIL2, 5 daily doses of 100,000 IU of IL-2, or vehicle. Shown are the study schema (**E**), tumor volumes (**F**), and body weight (**G**). **H-I**, NSG mice were implanted subcutaneously on the flank with $5x10^6$ A375 melanoma cells and tumors were allowed to establish for 11 days. On Day 0, mice received $3.8x10^6$ CAR-T cells intravenously. Mice received either vehicle or CD8-hIL2 (0.5 mg/kg) intravenously weekly for 3 weeks and tumor size was tracked over time. Shown are study schema (**H**) and tumor volume (**I**); the long arrow indicates injection of CAR-T on day 0 and the short arrows indicate injection of vehicle or CD8-hIL2 molecule. Data represented as mean ± s.d. and data are representative of 2 independent experiments. CR = complete response.
