# Supplementary material for: IL2 Targeted to CD8+ T Cells Promotes Robust Effector T-cell Responses and Potent Antitumor Immunity
Source: Cancer Discov. 2024 Apr 9;14(7):1206–25. doi: 10.1158/2159-8290.CD-23-1266 (PMC11215410; doi:10.1158/2159-8290.CD-23-1266)
Supplement: Supplementary Figure S5 — Anti-tumor activity of CD8-mIL2 in combination with anti-PD-1 in B16F10, 1956, MCA-205, and KP.mLama4 tumor models. [file cd-23-1266_supplementary_figure_s5_suppsf5.pdf]

Supplementary Figure S5

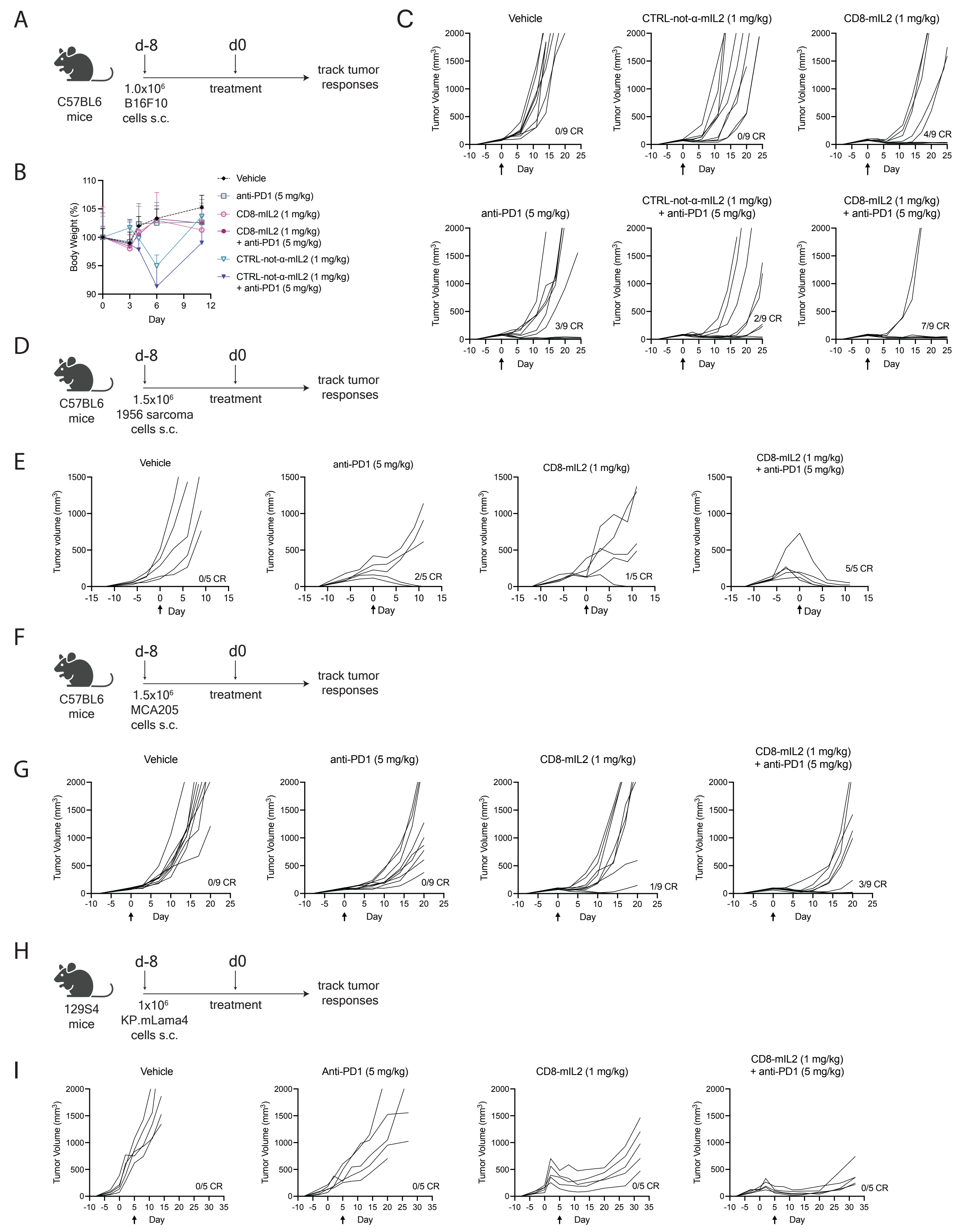

Supplementary Figure S5: Anti-tumor activity of CD8-mIL2 in combination with anti-PD-1 in B16F10, 1956, MCA-205, and KP.mLama4 tumor models. A-C, C57BL6 mice were implanted with B16F10 cells s.c. and treated 8 days later with a single dose of 1 mg/kg of CTRL-not-α-mIL2 or CD8-mIL2 as monotherapy or in combination with 5 mg/kg of anti-PD-1. Shown are the study schematic **A**, body weight **B**, and tumor volume **C** (n=9, representative of 3 independent experiments). **D-E**, C57BL6 mice were implanted with 1956 sarcoma cells s.c. and treated 8 days later with 1 mg/kg of CD8-mIL2 as monotherapy or in combination with 5 mg/kg of anti-PD-1. Shown are the study schematic, **D** and tumor volume, **E** (n=5). **F-G**, C57BL6 mice were implanted with MCA205 cells s.c. and treated 8 days later with 1 mg/kg of CD8-mIL2 as monotherapy or in combination with 5 mg/kg of anti-PD-1. Shown are the study schematic, **F** and tumor volume, **G** (n=9). **H-I**, 129S4 mice were implanted with $1x10^6$ KP.mLama4 tumor cells s.c. and were treated 8 days later with a single dose 1 mg/kg of CD8-mIL2 as monotherapy or in combination with 5 mg/kg of anti-PD-1. Shown are the study schematic, **H**, and tumor volume, **I** (n=5). Data in **B** represented as mean ± s.d.; CR = complete response.
