# Supplementary material for: IL2 Targeted to CD8+ T Cells Promotes Robust Effector T-cell Responses and Potent Antitumor Immunity
Source: Cancer Discov. 2024 Apr 9;14(7):1206–25. doi: 10.1158/2159-8290.CD-23-1266 (PMC11215410; doi:10.1158/2159-8290.CD-23-1266)
Supplement: Supplementary Figure S6 — Characterization of antigen-specific CD8+ T cells in MC38 tumors. [file cd-23-1266_supplementary_figure_s6_suppsf6.pdf]

Supplementary Figure S6

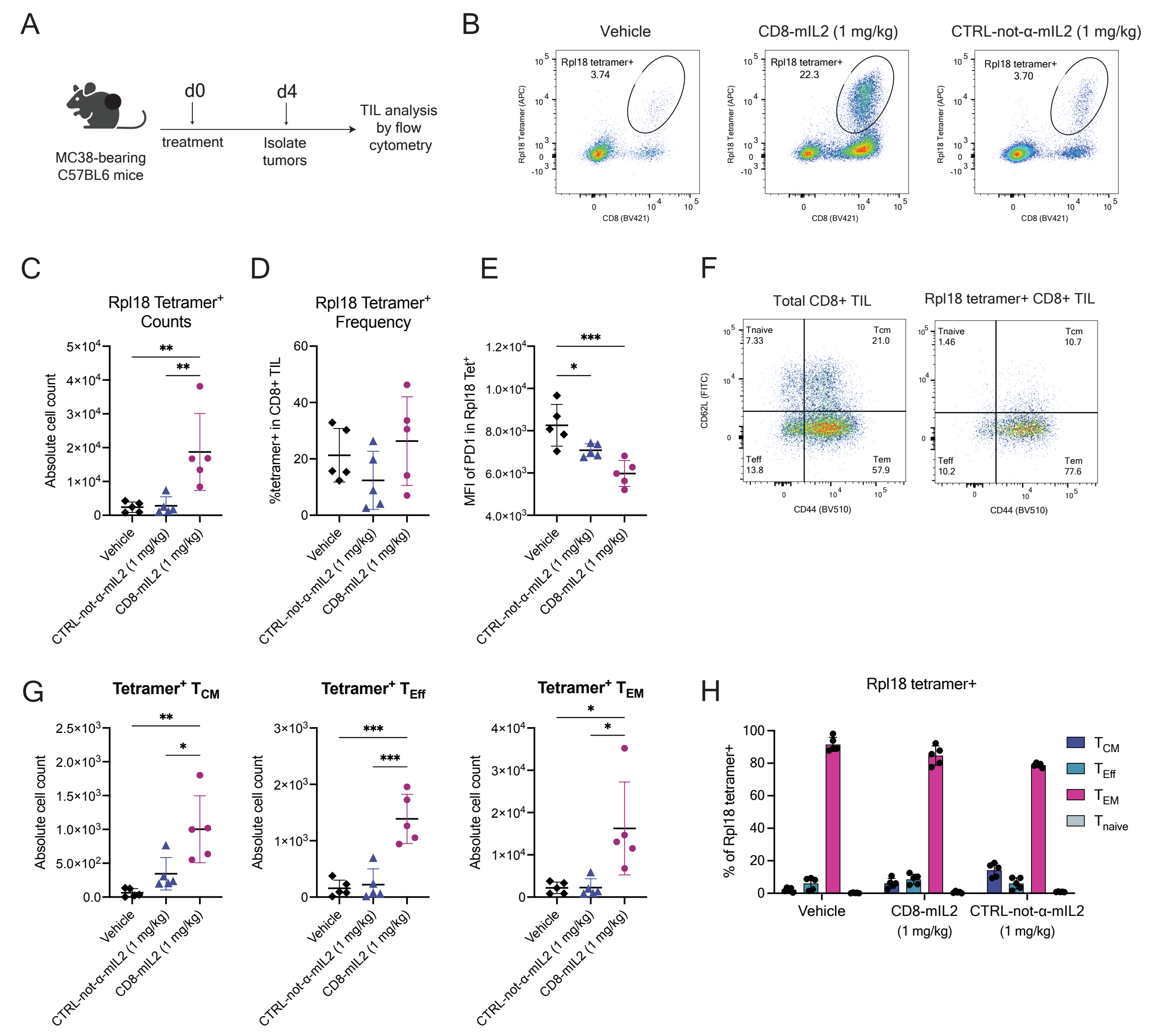

Supplementary Figure S6: Characterization of antigen-specific CD8⁺ T cells in MC38 tumors. A-G, MC38-bearing C57BL6 mice were treated with CD8-mIL2 or CTRL-not-α-mIL2 at 1 mg/kg and tumors were isolated and analyzed via flow cytometry. Shown are the study schema, **A**, and representative Rpl18 tetramer staining **B**. Also shown are the absolute count, **C**, and frequency, **D**, of Rpl18 tetramer$^+$ CD8$^+$ T cells, and **E**, the MFI of PD-1 on Rpl18 tetramer$^+$ CD8$^+$ T cells. **F,** Representative flow staining for identification of CD8+ T cell effector/memory subsets. **G**, Counts of T_{CM}, T_{EFF}, and T_{EM} tetramer$^+$ CD8$^+$ T cells, and **H**, the percentage breakdown by effector/memory subset of Rpl18 tetramer$^+$ CD8$^+$ T cells. Data in **C-E**, **G**, and **H** are represented as mean ± s.d.; statistics performed via one-way ANOVA with Dunnett's multiple comparisons test (n.s., P > 0.05, *P < 0.05, **P < 0.01, ***P < 0.001, ****P < 0.0001).
