# Supplementary material for: IL2 Targeted to CD8+ T Cells Promotes Robust Effector T-cell Responses and Potent Antitumor Immunity
Source: Cancer Discov. 2024 Apr 9;14(7):1206–25. doi: 10.1158/2159-8290.CD-23-1266 (PMC11215410; doi:10.1158/2159-8290.CD-23-1266)
Supplement: Supplementary Figure S7 — Sub-clustering of naïve-like/recently activated cluster and characterization of clonal expansion and antigen. [file cd-23-1266_supplementary_figure_s7_suppsf7.pdf]

# Supplementary Figure S7

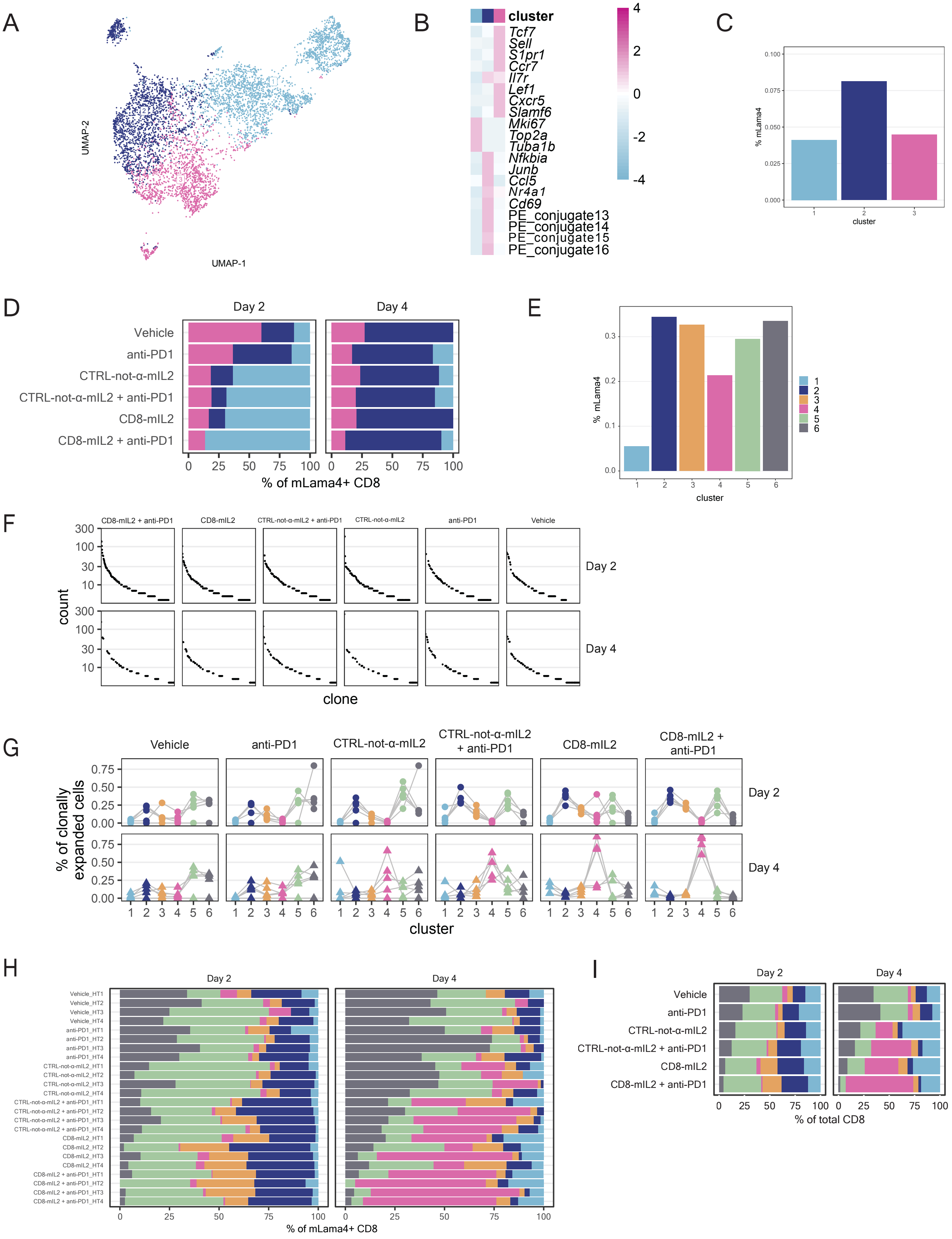

**Supplementary Figure S7: Sub-clustering of naïve-like/recently activated cluster and characterization of clonal expansion and antigen.** Shown are **A,** UMAP visualization of naïve-like/recently activated $CD8^+$ T cells according to cluster, **B**, relative expression (z-score of cluster average log-normalized counts) of selected genes across naïve-like/recently activated clusters. PE_conjugate refers to BEAM reagent labeling mLama4-reactive T cells. **C**, the percentage mLama4-specific $CD8^+$ TILs, by naïve-like/recently activated cluster, and **D**, proportion of mLama4-specific $CD8^+$ TILs in each cluster on day 2 and day 4 after therapy in each treatment condition. **E**, the proportion of mLama4-specific $CD8^+$ TILs in each cluster on day 2 and day 4 after therapy in individual mice. **F,** the percentage mLama4-specific $CD8^+$ TILs, by $CD8^+$ cluster. **G**, the proportion of clonally expanded $CD8^+$ TILs, defined as detection of 3 or more cells with a given TCR sequence, in each cluster on day 2 and day 4 in each treatment condition. **H**, the number of $CD8^+$ TILs in each clonally expanded clone by treatment/day, and **I**, the proportion of pan-$CD8^+$ TILs in each cluster on day 2 and day 4 after therapy by treatment condition. Colors used in **E**, **G**, **H,** and **I** correspond to the clusters as identified in **Fig. 6B**.
