# Supplementary material for: IL2 Targeted to CD8+ T Cells Promotes Robust Effector T-cell Responses and Potent Antitumor Immunity
Source: Cancer Discov. 2024 Apr 9;14(7):1206–25. doi: 10.1158/2159-8290.CD-23-1266 (PMC11215410; doi:10.1158/2159-8290.CD-23-1266)
Supplement: Supplementary Figure S8 — Differential expression by treatment. [file cd-23-1266_supplementary_figure_s8_suppsf8.pdf]

Supplementary Figure S8

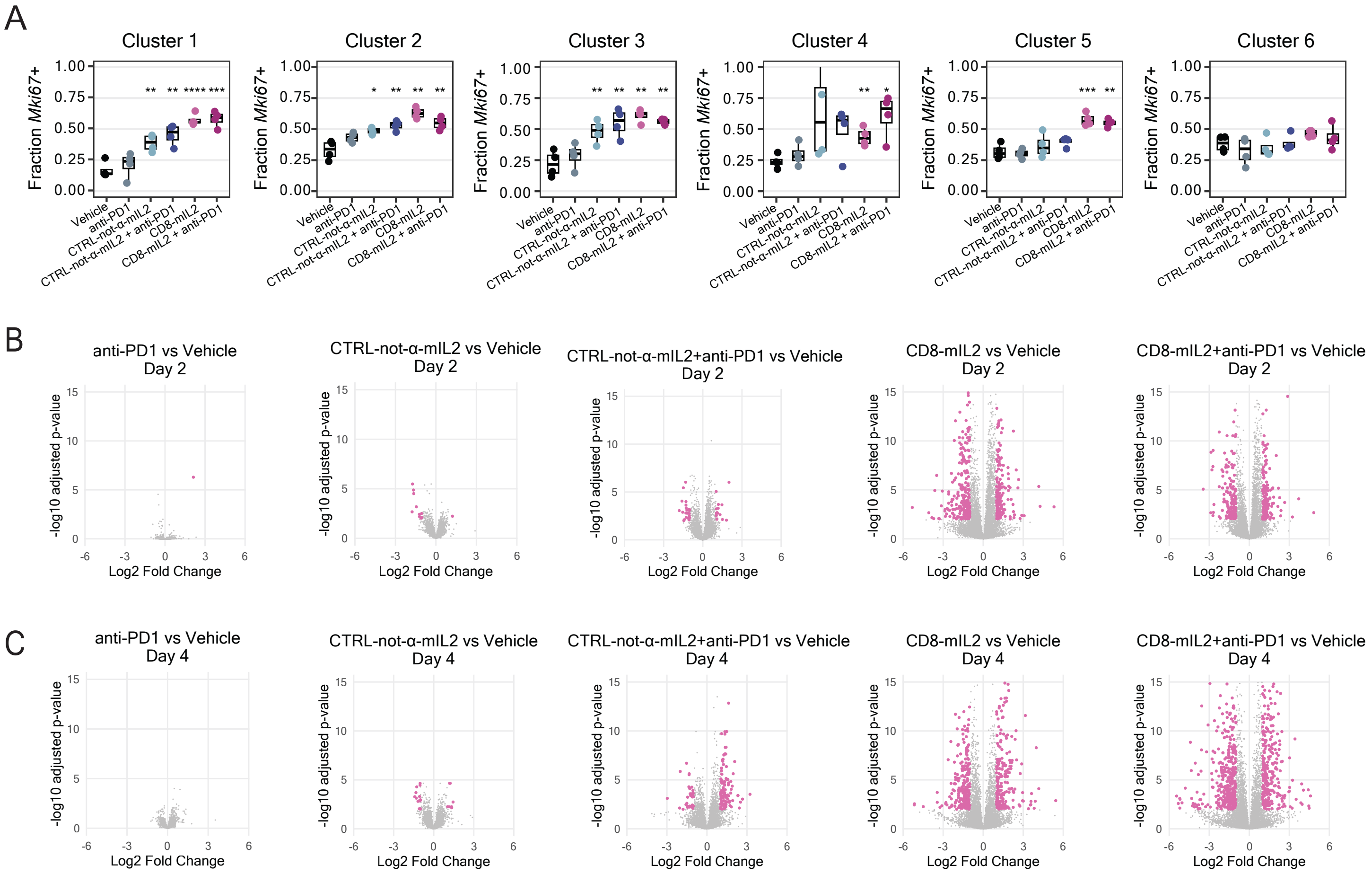

Supplementary Figure S8: Differential expression by treatment. Percent *Mki67+* positive by treatment condition and CD8$^+$ TIL cluster in mLama4-specific CD8$^+$ TILs at day 2 (**A**). Volcano plots for differentially expressed genes vs. vehicle control in mLama4-reactive CD8$^+$ TILs at day 2 (**B**) and day 4 after treatment (**C**) for the indicated treatment groups.
