# Supplementary material for: IL2 Targeted to CD8+ T Cells Promotes Robust Effector T-cell Responses and Potent Antitumor Immunity
Source: Cancer Discov. 2024 Apr 9;14(7):1206–25. doi: 10.1158/2159-8290.CD-23-1266 (PMC11215410; doi:10.1158/2159-8290.CD-23-1266)
Supplement: Supplementary Figure S9 — scRNAseq analysis of MC38 tumors. [file cd-23-1266_supplementary_figure_s9_suppsf9.pdf]

Supplementary Figure S9

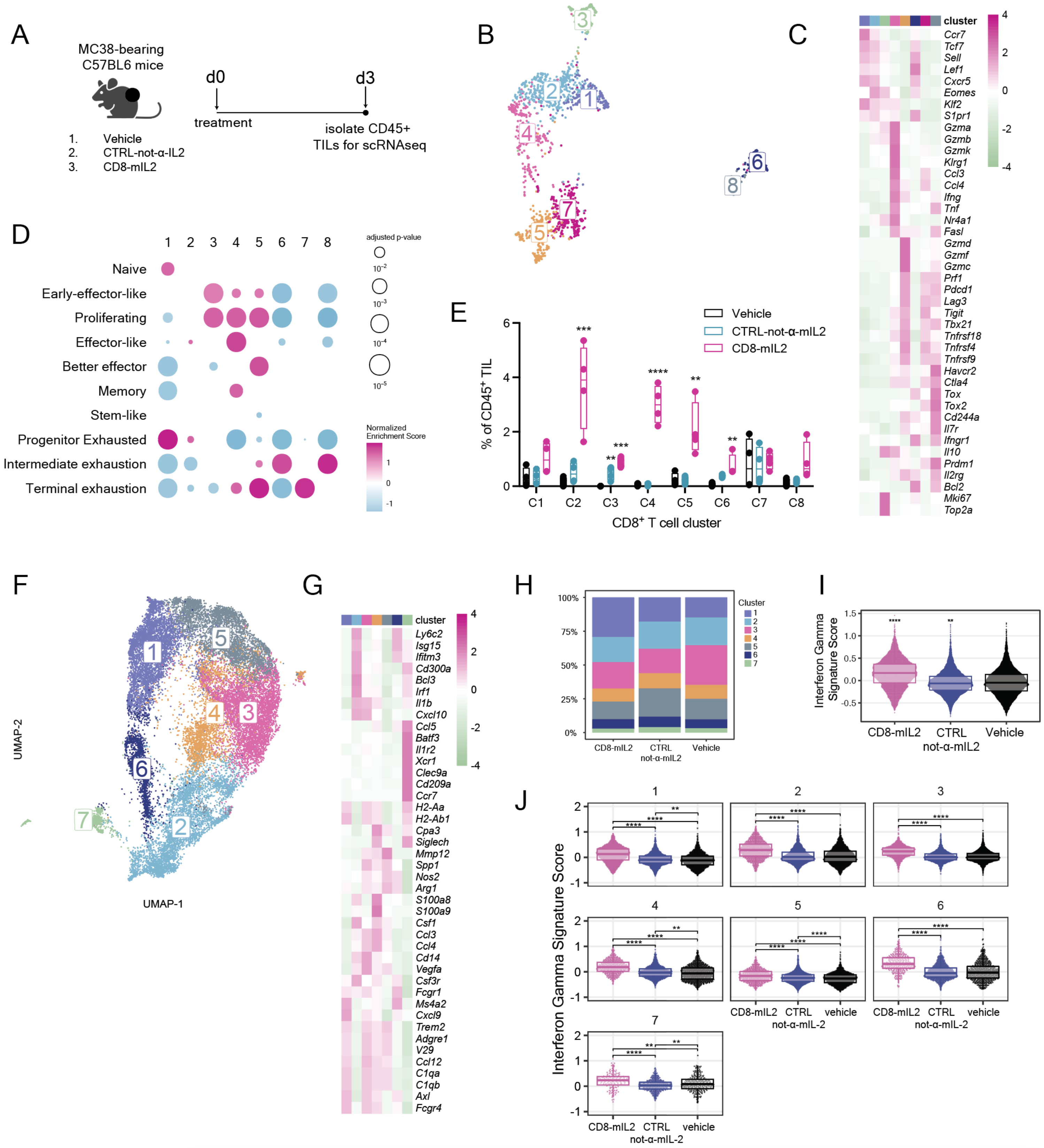

Supplementary Figure S9: scRNAseq analysis of MC38 tumors. A, MC38 tumor-bearing C57BL6 mice were treated with vehicle or 0.5 mg/kg of either CD8-mIL2 or CTRL-not-α-mIL2. On day 3, $CD45^+$ TILs were sorted and analyzed by scRNAseq. **B-E**, analysis of $CD8^+$ TILs. **B**, UMAP visualization of $CD8^+$ TILs according to cluster. **C**, Relative expression (z-score of cluster average log-normalized counts) of selected genes across $CD8^+$ T cell clusters. **D**, Gene set enrichment analysis of differentially expressed genes per identified $CD8^+$ T cell cluster. **E**, Frequency of each cluster as a fraction of $CD45^+$ TILs by treatment. **F-J**, analysis of myeloid populations. **F**, UMAP visualization of myeloid populations according to cluster. **G**, Relative expression (z-score of cluster average log-normalized counts) of selected genes across myeloid clusters. **H**, frequencies of myeloid clusters by treatment. **I**, IFN-γ signature score within intratumoral myeloid cells across all clusters. In violin plots shown in **I-J**, each dot represents an individual cell. Statistics performed via one-way ANOVA with Dunnett's multiple comparisons test (n.s., $P > 0.05$, *$P < 0.05$, **$P < 0.01$, ***$P < 0.001$, ****$P < 0.0001$).
