# Supplementary material for: IL2 Targeted to CD8+ T Cells Promotes Robust Effector T-cell Responses and Potent Antitumor Immunity
Source: Cancer Discov. 2024 Apr 9;14(7):1206–25. doi: 10.1158/2159-8290.CD-23-1266 (PMC11215410; doi:10.1158/2159-8290.CD-23-1266)
Supplement: Supplementary Figure S10 — In vitro and in vivo activity of AB248 in cynomolgus monkey. [file cd-23-1266_supplementary_figure_s10_suppsf10.pdf]

Supplementary Figure S10

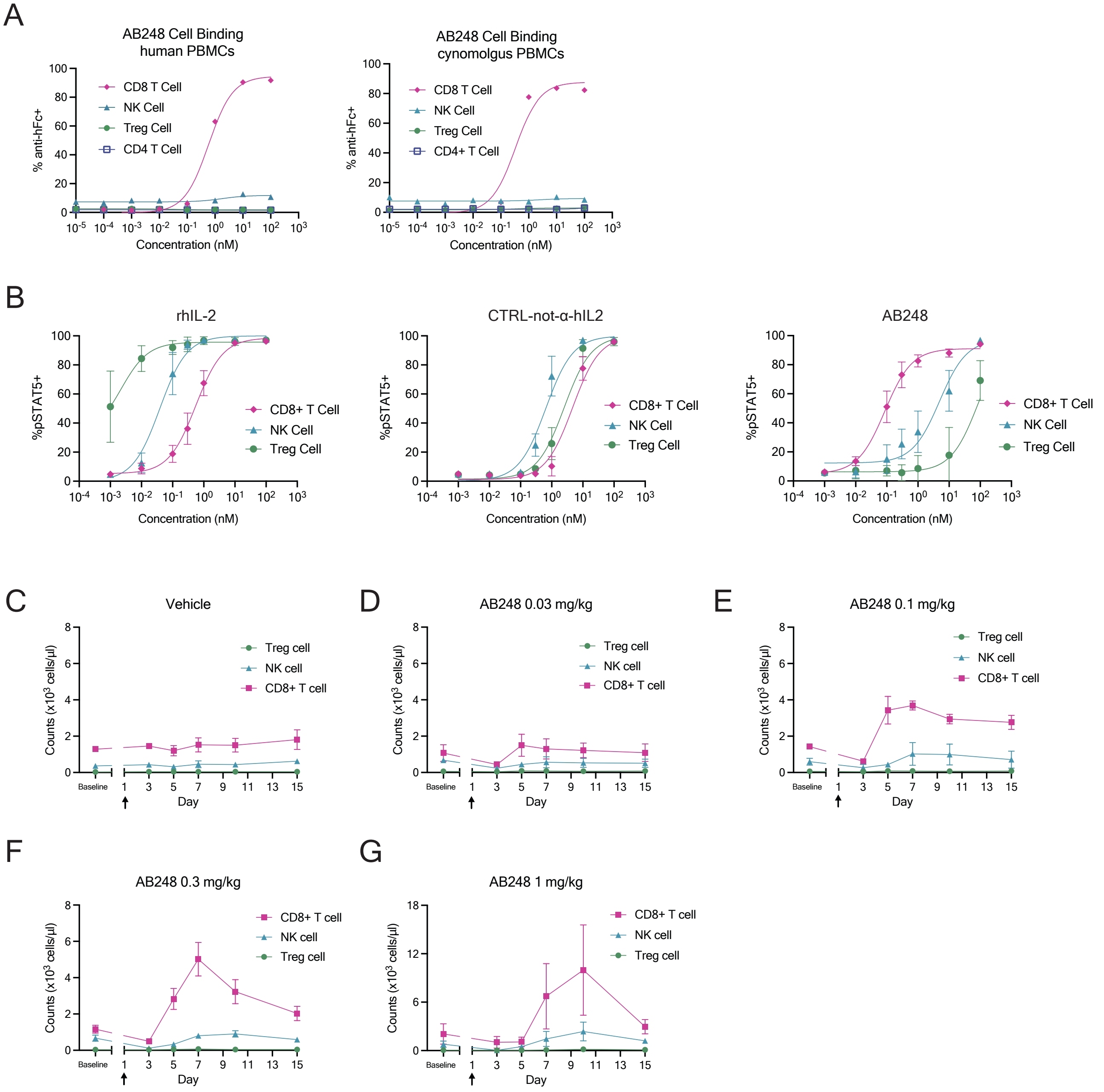

Supplementary Figure S10: In vitro and in vivo activity of AB248 in cynomolgus monkey. A, Human or cynomolgus PBMCs were incubated with AB248 for 2 hours at 4°C, washed, then stained with an anti-human Fc secondary antibody and analyzed for cell binding via flow cytometry. Shown is representative binding from human (left) or cynomolgus (right) PBMCs. **B**, pSTAT5 was assessed by flow cytometry following a 25-minute stimulation of cynomolgus blood with rhIL-2, CTRL-not-α-hIL2, or AB248 (n=5). Cynomolgus monkeys were dosed intravenously with vehicle (**C**) or AB248 at the indicated doses (**D-G**) and the indicated peripheral blood immune cell counts were assessed via flow cytometry and hematology (n=2-4 per dose level).
